# A versatile framework for resource-limited sentiment articulation, annotation, and analysis of short texts

**DOI:** 10.1371/journal.pone.0242050

**Published:** 2020-11-12

**Authors:** Vuk Batanović, Miloš Cvetanović, Boško Nikolić

**Affiliations:** 1 School of Electrical Engineering, University of Belgrade, Belgrade, Serbia; 2 Innovation Center of the School of Electrical Engineering, Belgrade, Serbia; Indiana University, UNITED STATES

## Abstract

Choosing a comprehensive and cost-effective way of articulating and annotating the sentiment of a text is not a trivial task, particularly when dealing with short texts, in which sentiment can be expressed through a wide variety of linguistic and rhetorical phenomena. This problem is especially conspicuous in resource-limited settings and languages, where design options are restricted either in terms of manpower and financial means required to produce appropriate sentiment analysis resources, or in terms of available language tools, or both. In this paper, we present a versatile approach to addressing this issue, based on multiple interpretations of sentiment labels that encode information regarding the polarity, subjectivity, and ambiguity of a text, as well as the presence of sarcasm or a mixture of sentiments. We demonstrate its use on Serbian, a resource-limited language, via the creation of a main sentiment analysis dataset focused on movie comments, and two smaller datasets belonging to the movie and book domains. In addition to measuring the quality of the annotation process, we propose a novel metric to validate its cost-effectiveness. Finally, the practicality of our approach is further validated by training, evaluating, and determining the optimal configurations of several different kinds of machine-learning models on a range of sentiment classification tasks using the produced dataset.

## Introduction

Sentiment analysis is one of the most popular and easily understandable and applicable tasks in the field of natural language processing (NLP). The general term *sentiment analysis* encompasses several specific subtasks, including polarity detection, subjectivity detection, sarcasm detection, etc. These tasks are often conceptualized in the form of binary classification problems, where the goal is to distinguish between positive and negative texts, subjective and objective texts, sarcastic and non-sarcastic texts, etc. Therefore, there have been many sentiment articulation schemes which tackle only one of these subtasks [1–6].

The most basic subtask is probably polarity detection [1, 2, 4]. The binary conception of this task is extremely simple, but surprisingly useful in real-world applications, such as social media monitoring or market research, when a general sentiment overview towards a certain topic is required [1, 7]. Nevertheless, enforcing a rigid binary separation can be problematic for texts that do not fall clearly in either category. This is why the complexity of sentiment articulation is frequently increased through one or more of the following means:

- Including a neutral class in the sentiment classification schema [8]–although the concept of a neutral class is seemingly quite intuitive, it is also somewhat unclear, due to the ambiguity of what statements should be considered neutral. As pointed out by Koppel and Schler [8], there are two conflicting ways of defining neutrality: one in which neutral texts express no sentiment at all (i.e. they contain only objective statements), and the other in which neutral texts include both positive and negative statements (i.e. neutrality is a mixture of sentiments). It is, of course, also possible to include both of these categories as separate classes in the classification scheme.
- Replacing the binary sentiment classification with a sentiment scale [9]–a sentiment scale makes it possible to assess not only the polarity of a sentiment, but also its strength. In systems that rely on a sentiment scale, the concept of sentiment neutrality is naturally represented via the average scores on that scale, but it is, again, unclear whether those scores should represent a mixture of sentiments or purely objective statements with no sentiment. A further complication in this annotation scheme is how to treat the mixture of sentiments if the central sentiment scale values represent statements with no sentiment and vice versa.
- Making sentiment judgments more fine-grained by assigning separate scores to different items in the text [10]–this approach, known as *aspect-based* sentiment analysis, makes it possible to articulate different sentiments towards different specific points within the text, rather than making a global judgment of the sentiment of the entire text. Aspect-based sentiment analysis seems, at first glance, to be an elegant solution to the issue of how to treat texts in which conflicting sentiments are expressed. For instance, if given the sentence *A was good*, *while B was bad*, an aspect-based approach would have a straightforward task of assigning a positive sentiment to A and a negative one to B. However, upon closer examination, it becomes evident that there are problematic situations in which moving to a finer grain does not provide any kind of relief. A good example of this phenomenon are texts which are inherently ambiguous, for instance: “*He’s right*, *you definitely haven’t seen something like this before*.”

Evidently, all of these sentiment articulation improvements have some issues of their own. Of course, it is possible to combine multiple sentiment scheme extensions in various manners, for instance, in the form of an aspect-based sentiment analysis approach that uses a sentiment scale. Still, even such relatively complex schemas cannot deal naturally with all ways of expressing sentiment in a natural language (e.g. the abovementioned ambiguous example remains a problem, there is still the question of how sarcastic texts should be marked, etc.). These issues are particularly apparent when dealing with short texts, in which sentiment can be strongly influenced by subtle figures of speech and slight variances in the tone of a statement. Creating a framework in which all such inherent intricacies of expressing sentiment could be noted and articulated in a direct manner would lead to a highly complex sentiment scheme. Such complexity would inevitably complicate the design, construction, and functioning of a sentiment analysis computational model. It would also make it quite difficult for humans to fully agree on the correct set of sentiment labels for a given text, thereby making the main goal of automatic sentiment analysis less clear.

Another especially important concern in the construction of sentiment analysis systems is the issue of resource availability. In this paper, by *resources* we refer to two distinct, but related points:

- NLP resources and tools, such as tokenizers, part-of-speech taggers, lemmatizers or stemmers, parsers, named entity recognizers, etc.–the existence and availability of these resources makes it possible to opt for more complex sentiment analysis models which rely on various types of text preprocessing and lower-level annotations.
- Financial and human resources required in the construction of a new sentiment analysis system for a particular language—manually annotated sentiment analysis datasets are necessary in model evaluation, as well as in supervised model training. Creating such datasets, according to an adopted schema and guidelines, requires human labor and expertise. The degree to which these factors are limited entails limitation with regard to the complexity and scope of the required annotations. Simpler annotation schemas necessitate fewer man-hours for the same amount of data and, similarly, require less time for annotator training, which is a necessary step in ensuring annotation consistency and quality.

Consequently, there is an inherent trade-off in the selection of a sentiment analysis scheme between detail and complexity on the one hand, and available resources on the other. Both kinds of abovementioned resources are typically not scarce when it comes to major languages, such as English or Chinese. Conversely, the creation of sentiment analysis systems for minor languages tends to be much more constrained, since languages with fewer speakers usually have less developed NLP resources and tools. Likewise, for most minor languages, it is more difficult to procure sufficient funding required to produce sentiment analysis (or, in general, any kind of NLP) datasets with appropriate manual annotations. Furthermore, crowdsourcing methods of dataset construction, which have proven to be a popular way of reducing costs when performing annotations in a major world language, are usually not a viable option for minor languages. This is due to the lower number of native speakers, which greatly amplifies the inherent difficulties in finding quality annotators in a crowdsourcing setup.

With these considerations in mind, it is clear that the usual approach of tackling each sentiment analysis subtask independently is ill-suited and cost prohibitive for resource-limited settings, as it requires separate dataset annotation efforts for each subtask, with an independent set of sentiment labels. In addition, the choice of the optimal computational model for sentiment analysis has to be constrained by the specific resource limitations at hand. This paper, therefore, focuses on three main research goals:

- Formulating a versatile and comprehensive framework for articulating and annotating the sentiment of short texts. The versatility of this framework is reflected in its sentiment labels having multiple possible interpretations, allowing a single annotation project to easily encode information relevant to multiple sentiment classification tasks.
- Verifying the cost-effectiveness of applying this framework, via a suitable cost-effectiveness metric.
- Determining the optimal configurations of sentiment analysis models within this framework, with a focus on models applicable in resource-limited settings.

The framework for short-text sentiment articulation, annotation, and analysis that we present in this paper is particularly suitable for resource-limited settings, since its sentiment labels simultaneously encode information regarding the polarity, subjectivity, and ambiguity of a text, as well as the presence of sarcasm or a mixture of sentiments. We demonstrate the cost-effectiveness of this framework on Serbian, a resource-poor but morphologically complex South Slavic language, via a novel method of evaluating the cost-effectiveness of a set of annotation guidelines. In doing so, we also present the first publicly available sentiment analysis corpora of short texts in Serbian. We decided to focus on movie comments, since the movie domain is typically the most difficult one to deal with in sentiment analysis [2]. However, we took great care to make our approach as domain-independent as possible, and we therefore also demonstrate its applicability to another domain—book comments. We subsequently train and evaluate different families of machine-learning classifiers on the annotated data in order to validate the practicality of our approach in terms of automatic sentiment classification, and to determine the optimal modeling options in these settings.

The remainder of this paper is structured as follows: in the next section, we give an overview of related work in sentiment analysis, with an emphasis on languages with limited resources. Afterwards, in the *Sentiment articulation and annotation* section, we present our sentiment articulation and annotation methodology, and we evaluate its quality, efficiency, and cost-effectiveness via a new cost-effectiveness metric. Subsequently, in the *Sentiment analysis* section, we examine the created sentiment analysis dataset in Serbian, we use it to train and evaluate several linear as well as neural state-of-the-art sentiment analysis models, and we discuss the results. The final section contains our conclusions and pointers regarding future work.

## Related work

Given the popularity and wide applicability of sentiment analysis, it is unsurprising that even a quick literature review offers thousands of approaches to addressing the task. For a general overview of the field we point to [11, 12]. Recently there has been a great deal of research in multilingual sentiment analysis [13–16], as well as in cross-lingual models [17–20]. Even though these developments may seem, at first glance, to obviate the resource limitations characteristic of minor languages, this is not actually the case, since manually annotated datasets in a particular language are still required to properly evaluate model performances on that language. Performing sentiment analysis by translating texts from a resource-limited language into a major one (e.g. English), be it manually or via machine translation, is also not a satisfactory solution, as translations of texts often do not preserve the original sentiment, due to both translation errors and cultural differences [21].

Quite a few distinct proposals of articulating text sentiment have been presented over the years, ranging from simple binary or ordinal classification schemas [1, 3, 4, 9], to multiclass sentiment annotation [22], to parse tree-based annotation [23], to complex annotation frameworks, often (partly) grounded in linguistic theories [24–26]. Existing short-text sentiment articulation schemas have mostly been developed for crowdsourcing annotations of Twitter data and thus tend to be as simple as possible, framing the annotation task in terms of determining whether a given sentence/tweet is positive, negative, or neutral [27], and relying on the annotators’ personal intuitions and interpretations of the task. In such frameworks, the common approach to dealing with particularly problematic texts, on which the annotators disagree and a majority vote is impossible, is to simply discard them (e.g. [28]).

As a result, so far not much attention has been given to the issue of choosing a suitable sentiment articulation schema and obtaining high-quality annotations for short texts under resource-constrained settings. The most relevant previous work with regard to such issues is the one by Mohammad [29]. In it, Mohammad identified several types of situations that make sentiment annotation difficult, including expressions in which different sentiments are expressed towards different targets of opinion, expressions of success/failure of one side versus another, sarcastic texts, quotations, rhetorical questions, etc. As a solution, Mohammad proposed two separate sentiment annotation schemas, one simpler and more appropriate for resource-limited situations, and another, more complex one. The simpler schema contains five distinct categories, based on the kind of language the speaker is using: a *positive* language category, a *negative* language category, a *sarcastic* language category, a category for *both positive and negative* language appearing in part, and a category for *neither positive nor negative* language. Apart from category descriptions and a few short notes, Mohammad did not provide any further guidelines regarding this schema, yet Mohammad et al. [30] did demonstrate its use through a crowdsourcing sentiment annotation of tweets in English. In this work, the authors merged the *negative* and the *sarcastic* language categories post-hoc, as well as the *both positive and negative* and the *neither positive nor negative* language categories. On this total of three classes, an inter-annotator agreement of 85.6% was reported.

There are also a few other papers which present short-text sentiment articulation schemas that could be applied in resource-limited settings. In the *Comparison with similar frameworks* subsection of this paper, we present a closer comparison between them and our own framework. Abdul-Mageed and Diab [22] proposed a four-class sentiment annotation schema designed for the news domain and applied it to a sentence-based annotation of a Modern Standard Arabic newswire sentiment dataset. They achieved an 88.06% agreement between two annotators, and a Kappa value of 0.823. The classes they employ are the following: Objective, Subjective-Positive, Subjective-Negative, and Subjective-Neutral. Their effort is significantly different to our own, since newswire texts are predominantly objective in nature, whereas the framework we present is more suited to texts without such inherent restrictions.

In a later paper [31], the same authors modified this schema by dropping the Objective category, introducing the Mixed sentiment category, and extending the annotation guidelines with a few additional points, including instructions based on politeness theory [32] and those regarding the treatment of agreements/disagreements. They applied this modified approach to other domains—Arabic Wikipedia talk pages and Arabic web forums, obtaining Kappa values of 0.790 and 0.793, respectively. They also experimented with simple guideline-lean three-class sentiment annotation (positive/negative/neutral), both with and without crowdsourcing, but they obtained very poor agreements between such annotation and their own gold labels (Kappa values ranging from 0.065 for crowdsourced data to 0.19 for traditionally annotated data).

Al-Twairesh et al. [33] used a five-class sentiment schema, which included the positive, negative, mixed, neutral, and indeterminate class, to annotate a corpus of tweets in the Saudi dialect of Arabic. They also presented a set of seven short annotation guidelines, with which the annotators familiarized themselves during a one-hour training session. They achieved a moderate level of annotation agreement between their three annotators, with a Kappa value of 0.60.

Unfortunately, none of the work presented above gives any information regarding the time it took their annotators to complete the sentiment-labeling task, making it impossible to measure the cost-effectiveness of the proposed approaches. To the best of our knowledge, Balamurali et al. [34] are the only ones who presented any kind of cost-benefit analysis of sentiment annotation, specifically regarding the issue of annotating and afterwards using WordNet senses rather than plain words as features for sentiment classification. However, their work considered only the basic positive/negative polarity classification. Joshi et al. [35] proposed a measure of sentiment annotation complexity, but their approach relies on eye-tracking gold label data, which is usually not available, as well as a set of linguistic features of the annotated text, many of which (e.g. coreference distance) are impossible to obtain automatically in low-resource languages.

With regard to sentiment analysis in the Serbian language, little work has been done so far. Batanović et al. [36] presented the first publicly available balanced sentiment analysis dataset in Serbian, consisting of document-level movie reviews separated into three classes—positive, negative, and neutral—and evaluated several morphological normalization methods for Serbian on it [37, 38]. Mozetič et al. [14] analyzed the effects of annotator agreement on the performance of machine-learning classifiers on the task of multilingual Twitter sentiment classification. They used datasets in 13 European languages, one of which was Serbian, and three sentiment classes—positive, negative, and neutral—but they found the Serbian data to suffer from quite low inter-annotator agreements. More recently, Ljajić and Marovac [39] evaluated different ways of handling negation in Serbian, using a corpus of tweets divided into the positive, negative, and neutral class, but they did not discuss what—if any—annotation guidelines they followed and the resulting dataset was not made public.

In light of this previous body of work, we endeavored to create a framework for sentiment articulation, annotation, and analysis that would be both applicable to various domains, as well as cost-effective and suitable to low-resource settings. With regard to the Serbian language in particular, we aimed to apply such a framework to produce a high-quality publicly available short-text sentiment analysis dataset.

## Sentiment articulation and annotation

In this section, we present the sentiment articulation and annotation scheme that we developed on and applied to *SentiComments*.*SR*, a corpus of movie comments in Serbian. These comments were written by various visitors on *kakavfilm*.*com*, the largest movie review website in Serbian. The initial data gathering procedure resulted in an anonymized collection of 4660 comments. Each comment was assigned a unique ID based on the movie to which it referred, and its placement in the comment tree for that particular movie, signifying its position within the whole discussion. A significant number of comments were too long to be considered short texts, so we disregarded all comments containing more than a predefined upper bound for token count (using basic whitespace tokenization). We also ignored several comments that were not written in the Serbian language. These removals reduced the final comment count to 3490.

### Sentiment articulation

The developed sentiment articulation and annotation scheme consists of six sentiment labels, as follows:

- *+1* –for texts that are entirely or predominantly positive
*-1* –for texts that are entirely or predominantly negative
- *+M*–for texts that convey an ambiguous sentiment or a mixture of sentiments, but lean more towards the positive sentiment in a strict binary classification
*-M*–for texts that convey an ambiguous sentiment or a mixture of sentiments, but lean more towards the negative sentiment in a strict binary classification
- *+NS*–for texts that only contain non-sentiment-related statements, but still lean more towards the positive sentiment in a strict binary classification
*-NS*–for texts that only contain non-sentiment-related statements, but still lean more towards the negative sentiment in a strict binary classification

In addition to these labels, we also assigned a special marking to comments which we deemed sarcastic, by appending an *s* to the sentiment label. Since sarcasm always implies an expression of sentiment, the *s* marking can only be appended to *+*/*-1* and *+*/*-M* labels.

This sentiment articulation scheme was designed with versatility in mind, as it provides several straightforward ways of reducing the sentiment label set in subsequent processing:

- Reduction to pure polarity categorization, where only the sign of the label is considered.
- Reduction to subjective/objective categorization, where *+/-NS* labels represent objective texts, and the remaining four labels represent subjective texts.
- Reduction to a four-class categorization system, with a positive class (*+1*), a negative class (*-1*), a mixed/ambiguous class (*+/-M*), and a no sentiment class (*+/-NS*).
- Reduction to sarcasm detection, where only the presence or lack of the *s* marking is considered.

Such label adaptability is a very desirable trait in resource-limited settings, since it allows a single annotation effort to encode multiple layers of sentiment complexity, thereby increasing the value and applicability of the annotated data.

Starting from an initial set of annotation rules, comments from the corpus were manually annotated with regard to their sentiment by two annotators working together. We found such a setup to be necessary in order to devise annotation guidelines that are as clear and unambiguous as possible. In particular, we aimed to produce a set of detailed guidelines designed for a group of committed annotators, since we deem this to be the only realistic setup for obtaining high-quality annotations in minor languages.

Sentiment annotation was performed in four passes through the dataset. The first pass served for the annotators to familiarize themselves with various linguistic phenomena present within the data and to manually correct various typing errors and the lack of diacritics in some comments. The original texts were also saved in order to establish the extent to which text proofing affects sentiment analysis models. Manual proofing was preferred over automatic tools, since the texts in question belong to an informal register, with a large number of non-standard vocabulary items for which automatic tools would not be useful. The annotators did not make adjustments in cases where the same letter or a group of letters is repeated several times as a means of emphasizing a particular word, since this phenomenon can be of use in detecting an emotional charge in the text.

The second annotation pass covered those comments which clearly belonged to a particular category. Following the recommendation of Hovy and Lavid [40], the more problematic annotation decisions were left for the third pass. It was mostly within the third pass that guideline refinements were made, as a result of discussions and consultations between the annotators. The fourth pass served to verify the consistency of previous annotation decisions and to assign the *s* marking to those comments which we deemed sarcastic.

### Annotation principles

In order to ensure high annotation quality, we found it necessary to both establish clear general guidelines, and to explicitly deal with a number of frequent problematic situations. The full guidelines include multiple examples for each of the 21 annotation principles described in the following subsections.

#### Context

We decided to consider each comment by itself, with no recourse to the surrounding comments to provide additional context. Such an approach was necessary, since additional context was often not available due to the removal of long comments from the dataset, but it was also desirable from the standpoint of task and annotation simplification. Furthermore, in many real-world situations, the sequencing of textual comments bears no relation to their inter-relationship. To ensure that the wider context was not considered, comments were labeled with regard to their sentiment in a shuffled order. Although a context-agnostic approach to sentiment analysis is, generally speaking, susceptible to errors of text misunderstanding, we rarely encountered difficulties in confidently labeling the sentiment of a text without access to its context. Such difficulties mostly revolved around the uncertainty about whether a given comment is sarcastic.

#### Topical equality

We assigned equal weight in sentiment assessment to all parts of a comment, regardless of their topic. More specifically, we did not assign greater weight to statements regarding movies themselves than to statements dealing with other topics (e.g. movie reviews). This approach was adopted in order to ensure the generalizability of the annotation guidelines.

The only exception to this rule concerns the separation of reality-related discourse elements, which are predominant, from fictional ones, i.e. those that are related to the content of the movie (e.g. sentiment towards a particular fictional character). The latter were utilized in sentiment annotation only as possible indicators of sentiment towards reality-related items, since the plot of a bad movie can contain sympathetic characters or situations, while good movies often contain evil characters or depict tragic events. For instance, the following comment is unequivocally positive (label *+1*), even though it mentions the tragic fate of the main character: “*Odličan film*! *Majstorski odrađen*! *[…] Šta je sve jadna žena morala da trpi i da preživljava*! *[…] Vrhunski film*.” (“*An excellent movie*! *Masterfully done*! *[…] The things that poor woman had to put up with and go through*! *[…] A first-class movie*!”).

#### Sentiment compositionality

If a comment contained a number of separate statements, we used the principle of composite scoring. This means that each statement was evaluated by itself, while the final sentiment label was obtained by combining these partial labels.

If a comment contained both a statement expressing no sentiment as well as a personal opinion, the objective portion of the text was ignored when assigning the sentiment label. In other words, the presence of even a single opinionated statement within a longer comment excluded the *+/-NS* labels from consideration. As a result, comments which were assigned any of the other four sentiment labels may contain some objective statements within them, whereas comments labeled as *+/-NS* do not contain any opinionated statements.

The sentiment label *M* was used in cases where a comment contained separate sections expressing differing sentiments towards different aspects or via different viewpoints. One such example would be if one part of a comment praised the movie, but another criticized its review, where the polarity of the overall sentiment label *M* would depend on the more prominent sentiment. The same sentiment label was applied in cases where a distinction with regard to sentiment is made between a general stance towards an item and the stance towards a particular aspect of it. Typically, the more general stance would outweigh the particular one, as in “*Dobar film*.. *ali su efekti loši*” (“*Good movie*.. *but the effects are bad*”), which was assigned a *+M* label. However, the conjunction of several particular stances can outweigh the polarity of a single general one.

On the other hand, when a comment contained a mixture of sentiments towards a single aspect or item, but it was clear which sentiment is stronger, the sentiment label was assigned in accordance with the stronger sentiment (*+/-1*). Such situations often happen when the initial praise/criticism is marked as erroneous or irrelevant in a later part of the comment, or when an initial praise/criticism is negated and then reiterated even more strongly, thereby amplifying the intensity of the sentiment, as in the following example, labeled as *+1*: “*Skorseze nije talentovan reditelj*. *On je legenda*.” (“*Scorsese is not a talented director*. *He’s a legend*.”) Finally, the composition of several statements of the same polarity yielded the same polar label (*+/-1*) for the whole comment.

#### Sentiment duality

In some cases, a single statement may express both a positive and a negative sentiment directed towards different items or aspects. For instance, the comment “*pa da*, *samo što mislim da nikada nije dobio pažnju koju zaslužuje*” (“*Well*, *yeah*, *it’s just that I think it never got the attention it deserves*”) expresses both admiration towards the object of the statement (worthy of attention—positive sentiment) and regret that its value was not broadly recognized (negative sentiment). The sentiment label *M* was assigned in such cases, while its polarity was dependent on the more pronounced sentiment (in this example we assigned a negative polarity to the comment).

#### Sentiment strength

We did not distinguish between texts of a certain polarity based on the strength of the sentiment expressed within them. Consequently, two statements of the same polarity but of different strengths would be assigned the same sentiment label. For instance, both “*Remek delo*. *Tačka*!” (“*A masterpiece*. *Period*!*”*) and “*gledljiv je*. *slažem se sa recenzijom i ocenom*.” (“*it’s watchable*. *I agree with the review and the score*.”) would be labeled as *+1*. Similarly, the *M* label was assigned whenever diverging sentiments were detected, regardless of their respective strengths and the count of positive and negative statements within a comment. Numerical scores on a 1–10 scale were sometimes included within a comment, so we treated scores in the 1–4 range as negative, those in the 7–10 range as positive, while score values of 5 and 6 could indicate either polarity, depending on the textual content of a comment.

#### Statement authorship

With regard to sentiment annotation, we considered only those opinions whose author was the speaker himself/herself to be relevant. The viewpoints of other people mentioned in the comment were considered only if they indirectly revealed the stance of the speaker. Otherwise, they were treated as objective information, effectively ignoring them when considering the overall sentiment of the text. Therefore, a comment such as the following example would be labeled as *-1*: “*[…] čuo sam dosta dobrog o ovom filmu*, *ali po mom mišljenju nikako ne zaslužuje te hvalospeve [*…*]*” (“*[…] I’ve heard a lot of good things about this movie*, *but in my opinion it definitely doesn’t deserve the rave reviews […]*”).

This approach was taken since it is often the case that a speaker references other people’s opinions but does not share them. An approach which would treat those opinions equally to the speaker’s own would, for example, result in a comment being assigned a -*M* label due to someone else’s negative opinion, even though the speaker’s sentiment is entirely positive. Since sentiment analysis is primarily interested in the speaker’s own attitude towards something, such an alternative approach would not be desirable.

#### Comparisons

Comparisons were dealt with by first determining the item which is the main focus of a statement and the relationship between that discourse element and the item it is compared to. For example, if a comment discusses movie A (the item in focus), and an *A is better than B* / *B is worse than A* type of statement is encountered, where B is another movie, then such a comment would be labeled as positive, since the purpose of the comparison is to express the superiority of the item in focus over another item. Similarly, under these conditions, *A is worse than B* / *B is better than A* types of statements would be considered negative, since their purpose is to express the inferiority of the item in focus in relation to another item. This logic is, of course, equally applicable to all possible items or aspects, and not only to movies.

However, a comparison with the item in focus (A) might not be direct, but merely implied by mentioning and discussing another item (B). In such cases, B cannot be treated only in view of its role in the (implied) comparison and the sentiment expressed towards it must be taken into full account. Furthermore, it is not uncommon for commenters to not limit themselves to pure comparisons, but to include other sentiment-charged statements in the comment, as well. Both of these situations were dealt with by applying the aforementioned principle of compositionality. For instance, the following comment was assigned the *+M* label: “*OK je Contagion*, *ali realno je pomalo dosadan*. *Preporuka za Carriers*, *sličan film sa daleko manjim budžetom koji je na mene ostavio dosta jači utisak*.” (“*Contagion is OK*, *but a bit boring*, *to be honest*. *Would recommend Carriers*, *a similar movie with a way smaller budget which left a much stronger impression on me*.”).

#### Statements of agreement and disagreement

In the absence of other opinion indicators, statements of agreement with or support for some previously expressed opinion were labeled as *+M*, for instance: “*Amin od prve do poslednje reči*.” (“*Amen from the first to the last word*.”). However, in situations where the agreement/support is directed towards a clearly expressed idea/proposition, the sentiment can be unequivocally polar, such as in the following *+1* comment: “*slažem se sa ovom idejom o top listi*!” (“*I agree with this idea about a top list*!”).

In some rare cases, statements of agreement/support do not actually express any sentiment, but are purely fact-related, such as the following example, labeled as *-NS*: “*U pravu si*. *Sad sam proverio i video sam da podatak za budžet ipak nije tačan…*” (“*You’re right*. *I just checked and saw that the information about the budget isn’t actually correct…*”) Of course, there are many instances where, in addition to statements of agreement, a comment contains clearly expressed views of its author. In these cases, it was not necessary to base the sentiment label of the comment on the agreement itself.

Similarly to statements of support, statements of disagreement would, by themselves, be labeled as *-M* in our scheme. However, we found that virtually all statements of disagreement are accompanied by a clarification of the author’s stance which unambiguously determines the appropriate sentiment label.

#### Statements of confirmation and denial

Unlike statements of agreement and disagreement, confirmations and denials often refer to statements which do not express a sentiment. In such cases, in the absence of clearer opinion indicators, a *+NS* label was assigned to confirmations, and a *-NS* label to denials.

#### Ambiguous statements

Certain comments are ambiguous, i.e. their polarity can be either positive or negative depending on the broader context. Since this context was not taken into account during sentiment annotation, such comments were labeled as *M*. It should be noted that ambiguity resolution is not always possible even if the broader context is considered, due to the inherent ambiguity of language itself. For instance, the following comment is inherently ambiguous: “*Goran je u pravu*, *ovako nešto do sada sigurno niste gledali*.” (“*Goran’s right*, *you definitely haven’t seen something like this before*.”) The label polarity of ambiguous comments was chosen on the basis of the broader context that would be more likely or natural for the given comment.

#### Questions

Unless they contained a stance towards an item, questions were treated as statements without a sentiment and, hence, labeled as *NS*, since they generally do not convey either positive or negative sentiments. Questions that imply a degree of interest for a topic were labeled as *+NS*, while those that imply a hint of confusion or disbelief were labeled as *-NS*.

#### Quotations

Some comments contain quotations, which usually refer to the movie review that is the subject of the comment. Such quotations typically contain opinions and express sentiments whose author is not the commenter him/herself. These quotations were, therefore, handled in accordance with the aforementioned principle of statement authorship—effectively treating them as objective information unless the quotation was used to illustrate an opinion with which the commenter (dis)agrees. An exception to this rule are situations when it is clear that the author of the comment is also the author of the quote, i.e. situations when a speaker is referencing his/her earlier statement(s). In such cases, the sentiment of the quotation itself was also taken into account when determining the overall text sentiment.

#### Statements of intent, interest, wishes, pleas, requests, and suggestions

When considered on their own, expressions of intent, interest, wishes, pleas, requests, and suggestions were all labeled as *+NS*. At first glance, it might seem that an interest in or a desire/intent to do something carries a clearly positive connotation, but closer inspection reveals that such an approach to sentiment annotation would be problematic. For instance, the desire to watch a particular movie does not necessarily imply either that the comment author has formed a particular stance towards the movie in question, or that his/her stance towards it will be positive after watching it. Likewise, the cause of the intent to watch the movie does not necessarily have to be positive—e.g. it may stem from spite directed towards the broader reception of the movie. Of course, it is possible that the comment author expresses an opinion towards the object of an action before that action has been completed, but in such cases his/her stance has to be clearly formulated for it to be taken into consideration.

Similarly, an interest in a certain item does not necessarily imply that the stance towards the item is positive—for example, a person can be interested in war as a historical or social phenomenon, but that does not mean that he/she condones it. Since pleas, requests, and suggestions represent messages directed to another person with the aim of accomplishing certain wishes of the speaker, they were all treated in the same manner regarding sentiment annotation, and were marked as *+NS*.

#### Statements of courtesy

Pure statements of courtesy were generally labeled as *+NS* (e.g. expressions of gratitude) or–*NS* (e.g. apologies), since they represent a formality, and express neither a positive nor a negative sentiment.

#### Regrets

For the most part, expressions of regret indicate a clearly negative sentiment, and were therefore labeled as *-1*. However, on occasion, regrets can indirectly express a positive sentiment, such as when the speaker says that he/she would regret if something was not done or if something was missed.

#### Factual statements

The natural sentiment label for a comment which expresses factual information is *NS*. The polarity of that label depended on the sentiment that potentially lay behind the factual statement. For instance, the following factual statement implies a potentially positive sentiment towards a movie, and was thus labeled as *+NS*: “*Baš to*, *film je slojevit*, *mora se pažljivo gledati*, *inače se lako propusti poruka koju šalje*.” (“*Exactly*, *the movie has many layers*, *you have to watch it carefully or you’ll easily miss its message*.”).

#### Statement ordering

In some comments, both the positive and the negative polarity elements are equally numerous and strong, so the overall sentiment polarity depends on the ordering of statements within the comment. Generally, statements which come at a later point in the text have a slightly greater overall impact than those which come beforehand. For example, the overall label of the following comment would be -*M*: “*Pa to*, *zabavan jeste*, *ali je već viđen…*” (“*Right*, *it is fun*, *but it’s been done before…*”). An inverted statement ordering would, however, switch the polarity to *+M*: “*Pa to*, *već je viđen*, *ali jeste zabavan…*” (“*Right*, *it’s been done before*, *but it is fun…*”).

#### Statement tone

In some cases, the tone of a statement may reveal its sentiment, and this usually happens when the tone is negative. In such situations, comments that would otherwise be labeled *NS* can have their sentiment label switched to *-1*. For example, the last statement in the following comment reveals its negative sentiment and affects the label of the entire comment: “*Pisao sam samo jedan od tih tekstova*. *‘Body of Lies’ je čist akcioni film*, *ne pretenduje da bude ništa više od toga*. *Ti čitaš uopšte ko je koji tekst pisao*?” (“*I wrote only one of those texts*. *‘Body of lies’ is a pure action movie*, *it’s not trying to be anything more than that*. *Did you even pay attention to who wrote which text*?”).

Sometimes the tone of a statement is conveyed via an emphasis placed on certain words or portions of text, usually expressed through upper case capitalization. The repetition of punctuation signs is also occasionally used in this manner.

#### Laughter

The relationship between humor and sentiment is a complex one, since humor can be used to express both positive and negative sentiments (with negativity often conveyed via sarcasm), but it can also be employed without attachment to a particular sentiment or stance. Therefore, the representations of laughter in a comment, such as “*Hahaha*” or “*Hehe*”, when present with no clarification on what they refer to, were considered to express no sentiment and were labeled as +*NS*. Of course, in cases where it was clear that the laughter was an expression of joy or excitement, it was marked as a positive statement. Similarly, in cases when it was apparent that the laughter was derisive, a negative sentiment label was assigned to it.

#### Emoticons

In the absence of other sentiment indicators, emoticons were used in order to determine the sentiment of a text. For example, the comment “*tako sam i mislio*: */*” (“*I thought so*: */*”) would have been inscrutable with regard to its sentiment, if not for the emoticon which clearly indicates disappointment, i.e. a negative sentiment. However, we found many instances where the sentiment of a comment clearly differs from the sentiment of the emoticons used within it, signaling the risk in relying only on emoticons when determining sentiment labels [41].

#### Sarcasm

Sarcasm is a figure of speech by means of which the meaning of a text, and particularly its sentiment, differs greatly from the literal understanding of the text. By their very nature, sarcastic comments cannot be objective, since they always express the speaker’s opinion. Detecting sarcasm in written text is a difficult task even for humans, particularly when the surrounding context is absent. This is because almost any statement can, in theory, be sarcastic, since sarcasm is usually revealed via a statement’s tone. This information is much more apparent in verbal communication, and is often lost when considering a statement only in its written form. We did, however, find a few distinctive situations in which sarcasm can be evident in a written text:

- If the literal understanding of a comment is illogical or nonsensical, then it is almost certain that sarcasm is employed. A typical example of this is the following comment: “[…] *kako Vas nije sramota da unosite logiku i razum u ovu diskusiju*! *Znate vrlo dobro da tome ovde nije mesto*.” (“[…] *how dare you bring logic and reason into this discussion*! *You know very well they have no place here*.”)
- Some sarcastic comments are presented in the form of rhetorical questions, such as the following one: “*Je li sa par piksela više film odjednom dobar*?” (“*Does the movie suddenly become good with a few more pixels*?”)
- Sometimes, the sarcastic tone of a comment remains preserved even in the written form: “*Ako si mu presudio*. *Bravo za tebe*.:*)”* (“*You did well to judge it*. *Good for you*.:*)”)*

The aforementioned examples all express a negative opinion and were, therefore, labeled as *-1s*. Although a great majority of sarcastic comments are similar to these, we did encounter an example where sarcasm was used to express a favorable opinion (label *+1s*): “…*Poruka Pixaru*: *WALL-E*, *Up*, *Toy Story 3*, *pa dokle više*??? *Dajte*, *snimite neki osrednji film*, *da i drugi studiji imaju šansu*………:*)”* (“*… A message to Pixar*: *WALL-E*, *Up*, *Toy Story 3*, *where does it end*??? *Come on*, *make a mediocre movie*, *give the other studios a chance*………:*)”*). In rare cases where it was not entirely clear whether sarcasm was present, but it was very likely, the *+/-Ms* label was used. The sarcasm marking *s* was applied to all comments in which sarcasm was detected, even if only a portion of the comment text was sarcastic.

### Annotation quality

Since the main *SentiComments*.*SR* corpus was jointly labeled by two annotators, in order to assess the agreement between them we created and annotated two additional, smaller comment datasets. One of them, also focused on the movie domain, was constructed by gathering and combining visitor comments from two other movie review websites in Serbian–*gledajme*.*rs* and *happynovisad*.*com*. Because of our wish to verify the general applicability of our annotation guidelines, and given the availability of suitable online data in Serbian, we decided to construct another smaller dataset out of the collected book comments from the *happynovisad*.*com* website. The movie verification corpus contains 464 comments, while the one focused on books comprises 173 comments.

The two main annotators performed sentiment labeling on both verification corpora separately. In addition, we asked four other annotators to annotate the verification corpora as well, so as to measure the quality and clarity of the produced annotation instructions. All six annotators were graduate students, and none of the new annotators had previous experience in annotation. We gave the full annotation guidelines and examples to two of the new annotators. The remaining two were given only a brief explanation of the meanings of different sentiment classes in our sentiment articulation schema (as described in the beginning of the *Sentiment articulation and analysis* section of this paper), but no instructions on how to deal with various linguistic phenomena and problematic situations, forcing them to rely on their own personal intuition and understanding of the task. This was done in order to measure the inherent clarity of the sentiment articulation schema, as well as the usefulness and cost-effectiveness of using the guidelines we had compiled. Therefore, the six annotators were effectively divided into the following three groups:

1. The initial group (IG)–the original annotators who worked on the main movie comment corpus and were, thus, well acquainted with the annotation schema and guidelines.
2. The experimental group (EG)–the new annotators who were given the full annotation guidelines.
3. The control group (CG)–the new annotators who were given only a brief explanation of the meanings of different sentiment classes.

All groups annotated the movie verification corpus first, and then moved on to the book comment corpus. On both corpora, for each annotator pairing we calculate the intra-group pairwise percentage agreement scores, as well as Krippendorff’s alpha scores [42], as recommended by Artstein and Poesio [43]. For calculating the alpha score values, we utilize the Krippendorff Python library (https://github.com/pln-fing-udelar/fast-krippendorff). We measure the annotator agreements for multiple interpretations of our sentiment labels:

1. For pure polarity labeling, where we only look at the sign portion of a label (i.e. *+* vs—).
2. For subjectivity labeling, where we contrast the objective texts with the subjective ones (*+/-NS* vs. all the others).
3. For four-class sentiment labeling, where we divide the texts into clearly positive (*+1*), clearly negative (*-1*), mixed or ambiguous (*+/-M*), and those expressing no sentiment (*+/-NS*).
4. For the full six-class sentiment labeling, as described in our sentiment articulation schema.
5. For sarcasm labeling, where we contrast the sarcastic texts (i.e. those to whose label an *s* has been appended) with the non-sarcastic ones.

Tables 1 and 2 contain the intra-group pairwise agreement percentages and alpha scores for all these label interpretations and all three annotator groups on the movie and the book verification corpus, respectively. They also include inter-group alpha scores representing agreement levels between each pair of groups. Unlike simple agreement percentages, Krippendorff’s alpha scores take into account chance agreement, which is why the remainder of our discussion will be focused on them. Krippendorff [42] proposed two thresholds for interpreting the alpha values. He suggests that if *α* < 0.667, the agreement is not acceptable, if 0.667 ≤ *α* <0.8, the agreement is tentatively acceptable, while if *α* ≥ 0.8, the agreement is reliable.

**Table 1:** Annotator agreement percentages and Krippendorff’s alpha scores on the movie verification corpus.

Label interpretation	Intra-group pairwise agreements						Inter-group agreements		
	IG		EG		CG		IG & EG	IG & CG	EG & CG
	%	alpha	%	alpha	%	alpha	alpha	alpha	alpha
Polarity	0.966	0.929	0.933	0.861	0.948	0.887	0.895	0.874	0.857
Subjectivity	0.989	0.896	0.976	0.795	0.970	0.725	0.823	0.754	0.748
Four-class sentiment	0.955	0.934	0.873	0.814	0.815	0.697	0.853	0.724	0.721
Six-class sentiment	0.922	0.892	0.821	0.750	0.802	0.679	0.801	0.687	0.678
Sarcasm	0.991	0.829	0.983	0.628	0.974	0.131	0.658	0.391	0.396

**Table 2:** Annotator agreement percentages and Krippendorff’s alpha scores on the book verification corpus.

Label interpretation	Intra-group pairwise agreements						Inter-group agreements		
	IG		EG		CG		IG & EG	IG & CG	EG & CG
	%	alpha	%	alpha	%	alpha	alpha	alpha	alpha
Polarity	0.977	0.935	0.977	0.935	0.908	0.731	0.935	0.802	0.807
Subjectivity	0.971	0.929	0.954	0.889	0.838	0.520	0.880	0.661	0.625
Four-class sentiment	0.965	0.948	0.902	0.852	0.751	0.570	0.869	0.700	0.657
Six-class sentiment	0.948	0.924	0.884	0.832	0.711	0.517	0.848	0.664	0.623
Sarcasm	0.994	0.931	1.000	1.000	0.977	0.324	0.859	0.559	0.544

When considering the movie verification corpus, the highest scores overall are obtained, unsurprisingly, on the simplest task of polarity labeling. Here, the distinctions between the three annotator groups are relatively slight, and all groups achieve reliable (*α* ≥ 0.8) levels of agreement. As the label complexity increases, the agreement levels generally start to deteriorate. At the same time, the differences between the control group and the remaining two groups become more pronounced—they start to be evident on the subjective/objective label interpretation and culminate on the task of sarcasm labeling. The initial group consistently achieves the highest levels of agreement, above the reliability threshold for all label interpretations, which is to be expected given the more extensive experience of the annotators in this group, gained through working on the main comment corpus. The results of the experimental group are somewhat lower and mostly fall into the reliable or tentatively acceptable category, with the exception of sarcasm labeling, where the agreement levels are not acceptable. The control group usually achieves the lowest alpha scores, with simple polarity labeling being the only exception and the only case of a reliable agreement level for this group. The inter-group agreements are the highest between the initial and the experimental group and belong to the reliable range for all label interpretations except sarcasm labeling, which is to be expected, since both groups relied on the same set of detailed annotation instructions. With the exception of polarity labeling, agreements for the other group combinations are noticeably and consistently lower, highlighting the difference between the annotations produced by the control group annotators and those created by all of the other annotators, who followed the proposed annotation methodology.

The results on the book verification corpus follow the same general trends but, when compared to those obtained on the movie verification corpus, further demonstrate the significance of two effects: domain shift and experience accumulation. The agreement levels of the initial group remain relatively constant across the two corpora and are always within the reliable category. The control group, however, demonstrates significantly worse results on the book corpus, with unacceptable agreement levels on all label interpretations except polarity, indicating an adverse effect of domain shift on annotation efforts performed without comprehensive annotation guidelines. The experimental group, on the other hand, achieves notably higher scores on the book corpus, all of which belong to the reliable range of agreement values and which are sometimes on par with or even above those of the initial group. This demonstrates that our annotation schema and guidelines are not domain-tailored, but applicable to various domains. It also indicates that, within our framework, even new annotators can quickly, after covering only a few hundred texts, gain enough experience to reach the agreement levels of fully trained annotators, in spite of domain shifts. Similar patterns regarding inter-group agreements can be observed as on the movie verification corpus. In addition, on the book corpus, the agreement between the initial and the experimental groups is visibly higher than that of the other group combinations even on the task of polarity labeling. In fact, this pair of groups achieves reliable levels of agreement on all label interpretations on the book corpus.

### Annotation efficiency and cost-effectiveness

In order to demonstrate the cost-effectiveness of our approach, we monitored the time it took the annotators to complete the work on the verification corpora. Before doing so, the annotators in the experimental group were given a few hours to familiarize themselves with the annotation guidelines. As previously mentioned, all annotators were first given the larger movie verification corpus and then the smaller book verification corpus. Table 3 displays the averaged efficiencies of annotators in each group.

**Table 3:** Averaged efficiencies of annotators in each group.

Annotator group	Average length / speed of annotation	
	Movie verification corpus (464 comments)	Book verification corpus (173 comments)
IG	~6h / ~77 texts/h	~2h / ~87 texts/h
EG	~9h / ~52 texts/h	~3h / ~58 texts/h
CG	~3.5h / ~133 texts/h	~1.25h / ~138 texts/h

The annotators in the experimental group were, on average, around two and a half times slower than the ones in the control group. The initial group was around 50% faster than the experimental group, which indicates that, with more experience, the annotation guidelines can be successfully internalized, leading to faster annotator performance.

None of the previous papers of a similar kind provide information regarding the efficiency of their annotation efforts, making it impossible to compare the approaches in that regard. We feel that this is quite an important aspect of selecting a sentiment articulation schema under resource-constrained settings, and that defining a suitable comparison metric could be useful in choosing between several competing options. However, optimizing annotation efficiency should not come at the expense of annotation quality, which is why a cost-effectiveness metric should incorporate a measure of both.

As an attempt at heuristically defining such a metric, we propose that the cost-effectiveness of a set of annotation guidelines be measured as a simple ratio between the relative decrease in annotation disagreement levels and the relative decrease in annotation efficiency, both brought about by the use of said guidelines for a designated annotation scheme. A proposed set of guidelines should then be accepted if this ratio is higher than one, i.e. if the agreement improvements outweigh the efficiency reductions, and rejected otherwise. In this setup, the baseline would be annotation performed without instructions or guidelines. We call this proposed metric *Annotation Cost-Effectiveness* (*ACE*).

In order to measure the relative decrease in annotation disagreement levels, we could use Krippendorff’s alpha scores, as follows:

$$\Delta A_{1}=\frac{\left(1-\alpha_{CG})-(1-\alpha_{EG}\right)}{\left(1-\alpha_{CG}\right)}=\frac{\alpha_{EG}-\alpha_{CG}}{1-\alpha_{CG}}$$ (1)

where *α*_*CG*_ is the intra-group Krippendorff’s alpha score for the control group, i.e. the group of annotators that did not have a set of guidelines at their disposal, while *α*_*EG*_ is the intra-group alpha score for the experimental group, i.e. the group of annotators that had access to the proposed annotation guidelines.1−*α* is the level of chance-corrected annotation disagreement of a group of annotators (since 1 is the maximum value of alpha), making Δ*A*_*1*_ the relative reduction in annotation disagreement. The maximum of Δ*A*_*1*_ is also 1, and is achieved when *α*_*EG*_ = 1.

This measure of relative reduction in annotation disagreement considers only the theoretical maximum of one as the goal/upper floor for the agreement values. However, an increase in agreement levels which pushes them over the thresholds specified by Krippendorff [42] should be additionally rewarded when formulating an overall metric for expressing the relative reduction in annotation disagreements, since such an increase brings about a qualitatively different interpretation of annotation consistency. We therefore calculate a relative disagreement reduction with respect to each threshold, if the initial agreement level, i.e. the control group agreement, is lower than that threshold. While doing so, if the new agreement level, i.e. the experimental group agreement, is above the threshold, we use the threshold value as the upper bound, so as to limit the relative disagreement reduction to a maximum of one, as follows:

$$\Delta A_{0.8}=\frac{\text{min}\{\alpha_{EG},0.8\}-\alpha_{CG}}{0.8-\alpha_{CG}}$$ (2)

$$\Delta A_{0.667}=\frac{\text{min}\{\alpha_{EG},0.667\}-\alpha_{CG}}{0.667-\alpha_{CG}}$$ (3)

The total relative reduction in annotation disagreement is acquired as the mean of the relative reductions with regard to all goal/threshold values which are above the initial agreement, i.e. the control group agreement, as long as the experimental group agreement is higher than that of the control group. Otherwise, the overall metric is calculated only with respect to the theoretical maximum agreement value, since the concept of threshold crossing is introduced with the presumption of lower control group agreement values:

$$\Delta A=\{\begin{array}{ccc} \frac{\Delta A_{1}+\Delta A_{0.8}+\Delta A_{0.667}}{3}, & \alpha_{CG}\leq \alpha_{EG}, & \alpha_{CG}<0.667\\ \frac{\Delta A_{1}+\Delta A_{0.8}}{2}, & \alpha_{CG}\leq \alpha_{EG}, & 0.667\leq \alpha_{CG}<0.8\\ \Delta A_{1}, & \alpha_{CG}\leq \alpha_{EG}, & \alpha_{CG}\geq 0.8\\ \Delta A_{1}, & \alpha_{CG}>\alpha_{EG} & \end{array}$$ (4)

Measuring the relative decrease in annotation efficiency can be performed similarly:

$$\Delta E=\frac{S_{CG}-S_{EG}}{S_{CG}}$$ (5)

In (5), *S*_CG_ represents the measure of annotation speed for the control group, *S*_EG_ is the measure of annotation speed for the experimental group, and Δ*E* is the relative decrease in annotation efficiency. As the measure of annotation speed, we use the average number of texts annotated per hour. Of course, this implies that the texts being annotated are of a generally similar length, which is usually the case in organized annotation projects.

The final Annotation Cost-Effectiveness metric *ACE* is, hence, calculated as:

$$ACE=\frac{\Delta A}{\Delta E}$$ (6)

If *ACE* ≥ 1, then the use of the proposed set of annotation guidelines is cost-effective; otherwise, it is not. If *ACE* < 0, then the annotator agreement levels are most likely actually lower when guidelines are applied than when they are not (Δ*A* < 0 ⇒ *α*_*EG*_ < *α*_*CG*_). An alternative cause of such *ACE* values can theoretically be an increase in not only annotator agreement but also annotation speed (Δ*E* < 0 ⇒ *S*_*EG*_ > *S*_*CG*_), due to the usage of said guidelines. However, such a scenario is quite unlikely in practice. It is also mathematically conceivable for both of these options (Δ*A* < 0, Δ*E* < 0) to be true, in which case *ACE* would be greater than zero, but this kind of situation is very improbable in practice, as it would imply that the usage of guidelines lowers annotation agreement, but increases annotation speed.

The same *ACE* metric could also be used to compare two sets of annotation guidelines. To do so, all that is required is to simply treat the set which produces lower agreement levels as the baseline (i.e. the control group).

Krippendorff’s alpha score is quite versatile, being applicable not only to categorical annotation values, such as the ones in our work, but also to ordinal, interval, ratio, etc. It can also be used for measuring the agreement of multiple coders. Due to this, the proposed *ACE* metric should have wide applicability as well, both in terms of annotation task variety and the size of the annotator groups.

Using the formulas presented above, the intra-group alpha scores shown in Tables 1 and 2, and the averaged group efficiencies shown in Table 3, we calculated the *ACE* values for all interpretations of our annotation schema labels, and we present them in Table 4.

**Table 4:** Values of the proposed annotation cost-effectiveness metric *ACE* on the verification corpora.

Label interpretation	Movie verification corpus		Book verification corpus	
	IG vs CG	EG vs CG	IG vs CG	EG vs CG
Polarity	0.883	-0.378	2.379	1.517
Subjectivity	1.926	0.975	2.572	1.592
Four-class sentiment	2.116	1.138	2.597	1.527
Six-class sentiment	1.975	0.663	2.564	1.525
Sarcasm	2.219	1.227	2.614	1.725

As seen in the table, the only label interpretation on which the use of our guidelines has been counter-productive (*ACE* < 0, Δ*A* < 0) is the polarity annotation performed by the experimental group on the movie verification corpus. This is likely due to the limited experience the experimental group annotators had at that point, as well as the extreme simplicity of that task, which resulted in the guidelines not being cost-effective even for the initial group (*ACE* < 1). Using all other label interpretations, the initial annotator group achieves *ACE* values over 1 on the movie verification corpus. However, for the experimental group the proposed guidelines do not prove cost-effective with regard to six-class sentiment annotation on the same corpus, while the task of subjectivity annotation is on the very edge of cost-effectiveness. Such results are likely caused by the complexity of the annotation schema and the time necessary for the annotators to become fully accustomed to using it. The performances on the book verification corpus, which was annotated after the one belonging to the movie domain, validates these conclusions. On this corpus, cost-effectiveness was achieved by both the initial and the experimental group on all label interpretations. Furthermore, the consistent rise of *ACE* values for both groups when switching over to the book corpus demonstrates that domain shift makes the use of comprehensive guidelines even more cost-effective, across all label interpretations, and even on simple annotation tasks such as polarity labeling. The highest cost-effectiveness score is consistently obtained on sarcasm labeling, validating the difficulty of the task and the importance of instructions to guide the annotators through it.

### Comparison with similar frameworks

Although our sentiment articulation framework was specifically designed with low-resource settings in mind, the set of sentiment classes we used necessarily shares some similarities with previous annotation efforts. In this section of the paper, we endeavor to compare and contrast our approach to some prominent existing multiclass sentiment annotation efforts. As we will show, although general category names (e.g. *neutral*) are often used as a seemingly common point across different sentiment articulation systems, in reality they usually have a distinct scope and meaning within each system and are applied to markedly different kinds of statements, depending on the specific annotation principles.

The key differentiating trait of our framework is that it was designed with multiple label interpretations in mind. Some of the previous sentiment articulation approaches do allow for the possibility of merging certain sentiment classes in post-hoc processing (e.g. as was done with the schema of Mohammad [29] in [30]), but such occurrences are rare. Furthermore, the annotation guidelines used within these frameworks were not explicitly developed with this aim, making the validity of label reductions potentially questionable for some of the annotated texts.

The schema proposed by Mohammad [29] allows the annotators to easily deal with certain problematic expressions, but it still leaves them without any guidance regarding some difficult choices, e.g. how to deal with ambiguous statements, for which there is a clear label (*M*) in our approach. Moreover, Mohammad’s focus on the kind of language the speaker is using can be somewhat limiting, since we found that the sentiment of short texts often depends on factors beyond the vocabulary, such as a statement’s tone. In addition, although the attention to vocabulary does make the annotation task easier, we believe it can occasionally be misleading. For instance, Mohammad proposed that sport-related statements such as *A beat B* should be considered positive, and those like *A lost to B* negative, due to the identification of verbs in question as examples of positive or negative language. Nevertheless, it remains unclear how to deal with expressions such as *A defeated B* in this framework, since they are semantically identical to *A beat B*, but the verb *to defeat* (or, alternatively, the noun *defeat*) could legitimately be viewed as an example of negative language. In effect, there is a danger that the annotators could treat semantically identical statements differently with regard to sentiment annotation. In contrast, in our approach both statements would be labeled in the same manner, by following the comparison rule presented in the section on annotation principles. Nevertheless, our annotation guidelines are substantially longer than the ones presented by Mohammad, necessitating a longer training for annotators and making them ill-suited for crowdsourcing methods.

As mentioned, the framework of Abdul-Mageed and Diab [22] was designed with newswire texts in mind, so there are several prominent dissimilarities in how we treat some typical situations. Most importantly, while our approach is to consider only the sentiment of the speaker as relevant, Abdul-Mageed and Diab rely instead on the concept of a private state [44]–a state that is not subject to direct verification—which means that they also take into account the sentiments and opinions of people other than the text author. In other words, a text with positive private states would be classified into the Subjective-Positive category, regardless of whose private states they were. For instance, within their framework, the sentence “*Hopes for the release of hostages revived in the last 24 hours with the intervention of Libya*.” (examples taken from [22]) would be categorized as Subjective-Positive, whereas in our approach such a statement would be labeled as *+NS*, since it is merely reporting the sentiment of some other people, and the sentiment of the speaker remains unknown. A similar example for their Subjective-Negative category would be the following sentence, which would be labeled as–*NS* in our approach: *“A statement from the Turkish Foreign Ministry indicated that ‘Turkey follows with great concern the terrorist attacks that have occurred in recent days in Uzbekistan and Kyrgyzstan’”*.

Abdul-Mageed and Diab placed speculations about the future into the Subjective-Neutral category. Such statements would most often be labeled as *NS* in our approach, e.g. “*All indications are that this situation will not change after the elections*.” They did the same with commitments to propositions, whose label in our approach would depend on the sentiment expressed towards the proposition itself. Conversely, they also treated examples of arguing as belonging to the Subjective-Neutral class, such as the following sentence: “*I said*, *and I repeat it*, *the problem is not in crude oil but rather in oil derivatives*.” Given the clearly negative sentiment the author expressed towards oil derivatives, this statement would be labeled as *-1* in our approach. Abdul-Mageed and Diab also mention the role of illocutionary speech acts (e.g. expressives) in articulating sentiment. In our guidelines, we address these as well, with specific instructions for different sets of expressions.

In their later paper [31], the same authors extended their annotation guidelines with explicit instructions on dealing with agreements/disagreements and instructions based on politeness theory [32]. Their treatment of (dis)agreement is fundamentally different to ours, since they automatically annotate expressions of agreement/approval as positive, and those of disagreement/disapproval as either negative, in case of direct disagreement, or neutral, in case of indirect/softened disagreement. In our approach, on the other hand, agreements presented in the absence of other opinion indicators are labeled as *+M*, since we do not know the sentiment that the speaker agrees with. Under the same conditions, disagreements are labeled as–*M*. In some cases, even a *+/-NS* label would be the most appropriate one, if it is clear that the (dis)approval is purely fact-related. Of course, if the speaker (dis)agrees with a clearly expressed opinion, then the appropriate polar label (*+/-1*) is assigned on the basis of it. A further point of distinction between Abdul-Mageed and Diab’s and our own sentiment articulation framework lies in their reliance on politeness theory, which led them to label indirect and/or softened requests as positive, and direct requests as negative, whereas we would label requests, when considered on their own, as *+NS*.

Unlike our approach, in which ambiguous statements are labeled as *M*, the same as statements expressing a mixture of sentiments, Al-Twairesh et al. [33] assign ambiguous statements to the Indeterminate category, which is separate from their Mixed category. Furthermore, their guidelines state that if an emoticon of a polarity opposite to that of the remainder of the text appears, the text should be labeled as mixed. On the other hand, in our framework, this would not be an automatic decision, and would have to be evaluated on a case-by-case basis, since it is often the case that such discrepancies are indicative of sarcasm, and should be labeled accordingly. Al-Twairesh et al. also discuss some characteristic phenomena their annotators found hard to deal with. Some of them, such as quotes, are directly addressed in our approach, while others, such as supplications (also mentioned by Mohammad [29]), are not, since in our datasets we found supplications and addresses to God to be consistently used to express a negative sentiment (e.g. “*O bože pa dokle više s tim Titanikom*. *Ovo samo na brzo premotavanje može da se gleda*” (“*Oh*, *God*, *how much more of this Titanic*. *This is watchable only on fast forward*.”)).

## Sentiment analysis

In this section, we analyze the annotated dataset we created, and we use the main *SentiComments*.*SR* corpus to train and evaluate several machine learning-based classifiers. The practicality of our sentiment articulation framework is demonstrated by performing experiments for all of the five sentiment label interpretations: polarity detection, subjectivity detection, four-class sentiment classification, six-class sentiment classification, and sarcasm detection. In order to provide a thorough analysis of sentiment classification in these settings and determine the best model configurations, we also consider the effects of various text preprocessing options.

### Dataset analysis

**Fig 1:**
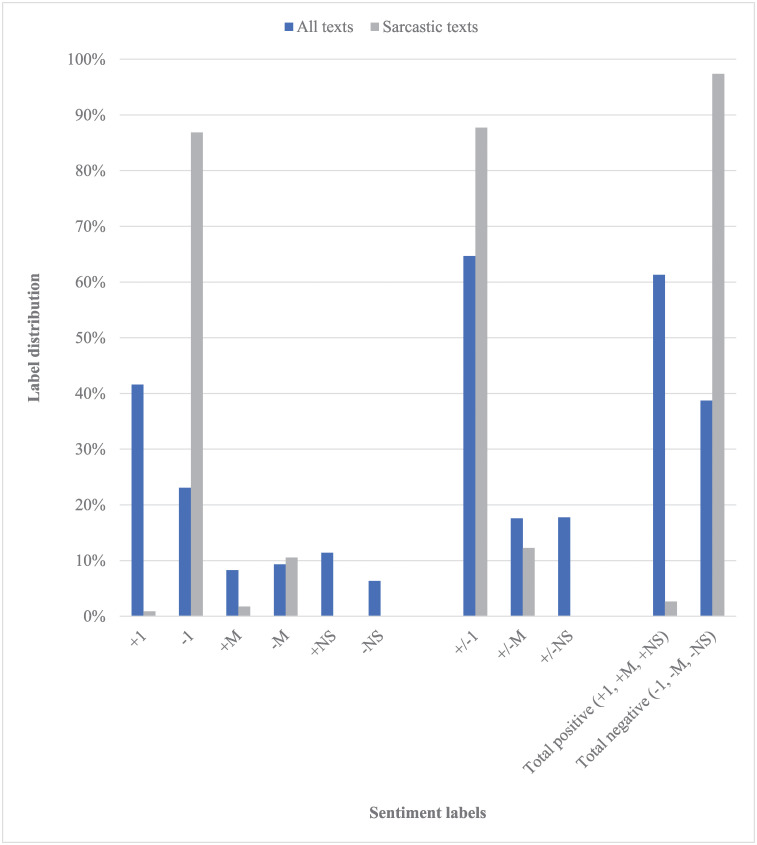
Distribution of texts in the main *SentiComments*.*SR* corpus across sentiment labels.

As mentioned, the main *SentiComments*.*SR* corpus contains 3490 short texts. The distribution of texts across sentiment labels is given in Fig 1 for both the entire corpus and its subset of sarcastic texts. The corpus is imbalanced, with an emphasis on polar texts, particularly positive ones (label *+1*). The prevalence of positive texts is also evident within the *NS* grouping, while the *M* labels are pretty equally distributed across the two polarities. The corpus contains 114 texts which were labeled as sarcastic, or around 3.27% of the total count. It is apparent that the vast majority of sarcastic comments are negative, which is to be expected. Similarly, most such comments are clearly polar, with around 10% of them being ambiguous/mixed.

S1 Appendix depicts and discusses the distribution of texts across sentiment labels for the movie and the book verification corpus. Because of their primary purpose with regard to measuring annotation quality, the verification corpora lack the unified, jointly agreed upon sentiment labels for the texts within them. Due to this reason, as well as the very small size of the corpora themselves, we do not use them in the training and evaluation of machine-learning classifiers. Instead, in the following evaluation sections we focus on the main *SentiComments*.*SR* corpus.

### Evaluation setup

Given the limited size of the *SentiComments*.*SR* dataset, we do not train data-hungry state-of-the-art algorithms from scratch, but rather focus on various linear models more suited to resource-limited settings, as well as pre-trained multilingual models, which are fine-tuned on our data. We also consider various text-preprocessing techniques that, though often simple, can have a noticeable impact on model performances, particularly when working with limited amounts of data.

Regarding general text preprocessing, we first transliterate all texts written in the Serbian Cyrillic script to their Latin script equivalents, since Serbian is a digraphic language. We then use the *ReLDI* tokenizer for Serbian, which is publicly available (http://reldi.spur.uzh.ch/blog/tokeniser/). Next, we evaluate the usefulness of manual text proofing, implemented during the sentiment annotation process. Finally, we experiment with two text normalization procedures. In the first one, repeated sequences of a single or multiple characters are normalized to a single occurrence, but they also trigger the insertion of a special “CHAR_REP” token. The motivation for using this procedure is the fact that character repetitions often convey the expression of an emotion. The second normalization deals with emoticons, by using a dictionary via which all emoticons are reduced to three classes—positive, negative, and ambiguous.

Since Serbian is a morphologically rich language, we consider the use of several morphological normalization methods, previously evaluated on other tasks, such as semantic similarity [45] or document-level sentiment analysis [36–38], to reduce data sparsity. These include four stemming algorithms implemented in the *SCStemmers* package [36]: the optimal and the greedy stemmer by Kešelj and Šipka [46], an improvement of their greedy algorithm proposed by Milošević [47], and a stemmer for Croatian, a closely related language, by Ljubešić and Pandžić, which is an improvement of the one presented in [48]. We also consider three lemmatization options. The first is the *BTagger*, available in two variants: one in which only word suffixes are normalized [49] and another in which full lemmatization is performed [50]. A lemmatizer for Croatian by Agić et al. [51] and a more recent lemmatization model for Serbian by Ljubešić et al. [52] are included in the evaluation, as well.

Furthermore, we examine a simple negation marking technique first proposed by Pang et al. [1] in which words after a negation word are prepended with a negation-marking prefix. Although rather basic, this method can be useful for languages and domains in which an adequate syntactic parser cannot be found. Since this is the case for the informal register of Serbian, which is predominant in our dataset, and since previous experiments on review-length documents [36] and tweets in Serbian [39] showed this approach to be beneficial, we explore its usefulness here, as well. In particular, we experiment with different scopes of negation marking, ranging from a single word after a negation to all the words between a negation and a punctuation symbol. In doing so, we follow the negation scope rules and patterns proposed by Ljajić and Marovac [39].

We begin our evaluation by using basic bag-of-words/n-grams (BOW) features and a set of linear classifiers implemented in the *Scikit-learn* library [53]. We consider the Multinomial (MNB) and the Complement Naïve Bayes (CNB) classifiers [54], logistic regression (LR), and a linear support vector machine (SVM), the latter two of which rely on the LIBLINEAR implementation [55]. For binary classification tasks we also consider NBSVM, a mixture of MNB and SVM that was shown to work well in binary settings [38, 56]. As suggested in [56], we employ the L2 regularization and loss function for SVM, NBSVM, and LR.

We then move on to a bag-of-embeddings setup, using a linear SVM classifier with features based on averaging the embeddings of words in each text. The embeddings are trained on the Serbian Web Corpus *srWac* [57], the largest publicly available corpus of texts in Serbian, containing 555 million tokens. Punctuation and words that are not in Serbian are removed from the *srWaC* corpus, which is then lowercased, reducing the corpus to around 470 million tokens and a vocabulary of around 3.8 million entries. We use the *word2vec skip-gram* algorithm [58, 59], as implemented in the *gensim* package [60] to generate embeddings, since preliminary experiments showed it to be consistently superior on these tasks to its alternative CBOW (*Continuous Bag-of-Words*) model. We also ran preliminary tests on the newer *fastText* algorithm [61] and found that its embeddings lead to quite similar classification performances as those produced by *word2vec*. Since *fastText* vectors were considerably slower to train, we opted for *word2vec* in subsequent experiments.

As the final part of the evaluation, we compare the aforementioned methods with newer, transformer-based ones. Specifically, we examine several multilingual/cross-lingual models which support Serbian, and we use the *HuggingFace Transformers* library’s [62] implementation. These models are: multilingual BERT [63], multilingual DistilBERT [64], and XLM [65]. We also experimented with XLM-RoBERTa [66], but did not include it in our comparison due to persistent convergence issues we encountered on our dataset. We interface with all these models using the *Simple Transformers* library (https://github.com/ThilinaRajapakse/simpletransformers).

All models are evaluated using 10-fold stratified cross-validation, with the weight-averaged F-measure as the performance metric, since the F-measure is often used in the field of sentiment analysis (e.g. [15, 26–28, 30, 33]). We opted for weight averaging rather than macro averaging since, as discussed in the section on sentiment articulation and annotation, the scope and meaning of each sentiment class is a matter of design, which is why we felt it was adequate for real-life class frequencies to have some impact on the overall performance score. The only exception is the sarcasm detection task where, due to a massive class imbalance, the F-measure for the sarcastic class is used as the performance metric.

### Evaluation results and discussion

We first present the results of models based on linear classifiers using bag-of-words and/or bag-of-embeddings features. Afterwards, we consider the results of the newer neural transformer-based methods, and we compare and discuss the performances of different model families.

#### Linear models

The evaluation of bag-of-words/n-grams models proceeds in the same manner across all tasks. We first examine the effects of basic text preprocessing options and then compare various morphological normalizers. Afterwards, we consider the impact of negation marking strategies, and finally consider term frequency/inverse document frequency (TFIDF) weighting and higher order n-gram features. For each set of settings, we consider the overall impact on all classifiers when selecting the optimal option. In particular, in order to avoid biasing our conclusions to a particular classification algorithm, we do not select as optimal any settings that have a positive effect only on some classifiers, yet a negative one on others. A nested 5-fold stratified cross-validation is used to optimize the SVM/NBSVM/LR cost hyperparameter C ∈ [10^−2^, 10^2^] and the *beta* ∈ {0.25, 0.5} hyperparameter of NBSVM, while the other hyperparameters are set to their default values. In all experiments, we lowercase the input texts.

Detailed evaluation results of bag-of-words models are shown in S2 Appendix. Overall, the NBSVM algorithm proves to be the optimal choice for polarity detection, while LR and SVM stand out in multiclass classification. Text proofing, character repetition and emoticon normalization, as well as morphological normalization, particularly the stemmer of Ljubešić and Pandžić, prove to be generally beneficial for BOW models on sentiment analysis tasks on our dataset, whereas the impact of negation marking is task-specific. The effects of TFIDF weighting are inconsistent, while the inclusion of higher order n-gram features is most often detrimental. We were able to successfully evaluate classifiers using all label interpretations, with the exception of sarcasm detection, where the very limited amount of sarcastic comments in the corpus prevented us from reaching confident conclusions regarding classifier performances. For this reason, we do not pursue further experiments regarding this task on our dataset.

We then move on to using averaged *word2vec skip-gram* embeddings as features for a linear SVM classifier with the same hyperparameter tuning settings as for the bag-of-words models. Detailed evaluation results of bag-of-embeddings models are shown in S3 Appendix. The results show that manual proofing, character repetition and emoticon normalization, and morphological normalization are, once more, beneficial to classification performances on all tasks, with Ljubešić and Pandžić’s stemmer proving to be the optimal choice in this setup, as well. Classification results also improve as the embedding dimensionality and window size are increased. Finally, combining bag-of-embeddings with bag-of-words features, particularly those in which negation marking is applied, outperforms either variant by itself. Table 5 contains the best results of bag-of-words, bag-of-embeddings, and joint models on four sentiment classification tasks.

**Table 5:** Best evaluation results of linear models.

Setting	Task results			
	Polarity	Subjectivity	Four-class	Six-class
Bag-of-words features	0.782	0.871	0.64	0.566
Bag-of-embeddings features	0.783	0.873	0.628	0.557
Bag-of-words + bag-of-embeddings features	0.783	**0.885**	**0.655**	**0.586**

#### Transformer-based models

Finally, we consider the following three transformer-based models that we fine-tune on our corpus for each task separately:

- Multilingual BERT [63]–we use the newer, cased version of the model, with 12 layers, 12 self-attention heads, and a dimensionality of 768. This model was originally trained on the top 104 languages with the largest Wikipedias, including Serbian.
- Multilingual DistilBERT [64]–a distilled version of the multilingual BERT model, with the same dimensionality and number of attention heads, but reduced to 6 layers. It supports the same languages as multilingual BERT.
- XLM [65]–we use the MLM (masked language modelling) version of XLM, with 16 layers, 16 self-attention heads, and a dimensionality of 1280, which was originally trained on 100 languages, including Serbian.

We begin our evaluation of these models by fine-tuning them for a single epoch for each task and comparing their performances on four variants of the *SentiComments*.*SR* corpus:

- The original, transliterated texts
- The corrected, manually proofed texts
- The corrected texts to which character repetition and emoticon normalization were applied
- The stemmed version of the corrected and normalized texts, obtained via the Ljubešić & Pandžić stemmer, which proved to be the optimal choice for morphological normalization in the previously considered setups

We use the default fine-tuning settings of the *Simple Transformers* library (batch size = 8, learning rate = 4e-5), and we retain text casing. The averaged results of five runs with different seeds are shown in Table 6. CR&EN in the table denotes the aforementioned character repetition and emoticon normalization procedure.

**Table 6:** Evaluation results of transformer-based models.

Model / Setting		Task results			
		Polarity	Subjectivity	Four-class	Six-class
*Epochs = 1*					
BERT Base Multilingual Cased	Original texts	0.725	0.862	0.538	0.493
	Corrected texts	0.735	**0.872**	**0.578**	0.497
	Corrected texts + CR&EN	**0.739**	0.867	0.573	**0.502**
	Stemmed texts + CR&EN	0.715	0.864	0.574	0.478
DistilBERT Base Multilingual Cased	Original texts	0.720	0.864	**0.548**	0.455
	Corrected texts	**0.725**	**0.869**	0.545	**0.465**
	Corrected texts + CR&EN	0.715	0.867	0.542	0.459
	Stemmed texts + CR&EN	0.713	0.857	0.538	0.451
XLM MLM	Original texts	0.739	0.873	0.634	0.553
	Corrected texts	**0.788**	0.873	**0.647**	**0.571**
	Corrected texts + CR&EN	0.779	**0.879**	0.646	0.547
	Stemmed texts + CR&EN	0.760	0.870	0.618	0.532
*Epochs = 3*					
Corrected texts					
BERT Base Multilingual Cased		0.785	0.879	0.635	0.604
DistilBERT Base Multilingual Cased		0.772	0.883	0.634	0.576
XLM MLM		**0.793**	**0.887**	**0.686**	**0.627**

We find that the models most often perform best on corrected, but un-normalized texts, which is unsurprising since this is the kind of text these models were trained on. In particular, the differences between the performances on the corrected and the normalized versions of the corpus are slight, but the application of stemming in this context is noticeably detrimental. Similarly, performances on the original texts are, for the most part, only slightly below those obtained on the corrected texts, but the differences do become more pronounced in certain model/task combinations.

Using the corrected texts as input, we then increase the length of fine-tuning to three epochs, as suggested by Devlin et al. [63], in order to compare the performances between the models. We find that the XLM model is the best-performing one on all tasks, and that the DistilBERT model performs quite closely to the original multilingual BERT. We also experimented with further increasing the number of epochs to five, but this yielded no consistent additional improvement.

## Discussion

**Fig 2:**
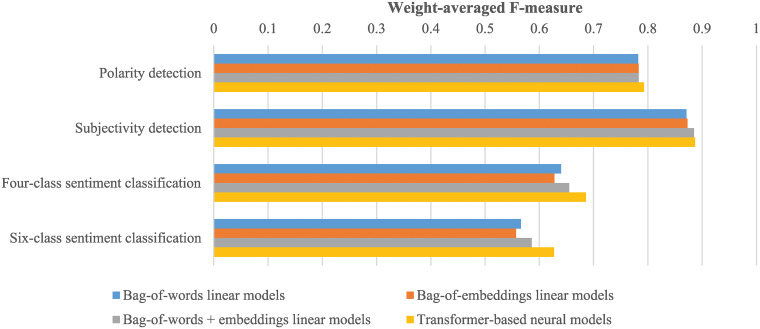
Comparison of the best results of different model families across various sentiment analysis tasks.

Several clear trends can be detected by reviewing the evaluation results we have presented. Fig 2 contains a graphical comparison between the best results of the different model families we have examined.

Firstly, we have established that linear bag-of-words models, coupled with simple text preprocessing techniques, still present a strong baseline in resource-limited settings. Bag-of-embeddings models tend to be, by themselves, equal to or slightly worse than BOW models, but the conjunction of these two kinds of features outperforms either single variant. Finally, we have demonstrated that fine-tuning multilingual transformer-based models leads to equal or better performances than the aforementioned linear classifiers. The differences between the model families are slight on the simpler binary tasks of polarity and subjectivity detection, and more pronounced on the more complex multiclass sentiment classification.

An added advantage of the transformer-based models is that they perform quite well without the preprocessing and normalization options we considered, making them easily applicable. However, such multilingual models still cover only a relatively small set of around a hundred languages. Falling back on the simpler models may, therefore, be a necessity for many minor languages. Nevertheless, as our results demonstrate, this downgrade may not lead to discernible performance penalties on the simpler binary classification tasks.

## Conclusion

In this paper, we have presented a framework for articulating, annotating, and analyzing the sentiment of short texts which is particularly suited to resource-limited settings, due to the versatility it provides with regard to sentiment label interpretation. We have compared and contrasted our framework with previous approaches, and demonstrated its use in the construction of *SentiComments*.*SR*, the first publicly available dataset of sentiment-annotated comments in Serbian. After discussing the quality and efficiency of our approach, its effectiveness was verified by applying a novel cost-effectiveness measure. We then trained and evaluated multiple machine-learning classifiers on the new dataset, ranging from linear bag-of-words and bag-of-embeddings models, to fine-tuned transformer-based neural architectures. The practicality of our sentiment articulation schema was validated by considering multiple sentiment classification tasks, using various interpretations of the overall sentiment labels. The evaluation also analyzed the effects of various model settings and text preprocessing options. The results demonstrate that simple bag-of-words models present a strong baseline within our framework, but that transformer-based models achieve state-of-the-art performances even in resource-limited settings.

In the future, we plan to apply our sentiment framework to create additional sentiment analysis datasets in other domains and languages, since we believe that our approach is well-suited for other resource-limited languages. We will also consider the creation of a monolingual transformer-based language model for Serbian and its subsequent use in downstream tasks. Finally, we intend to utilize our new cost-effectiveness metric in further annotation efforts, particularly those in which a choice between multiple annotation strategies is required.

## Supporting information

S1 Appendix Distribution of texts in the *SentiComments*.*SR* verification corpora across sentiment labels. (DOCX)

S2 Appendix Detailed sentiment analysis evaluation results of bag-of-words classifiers. (DOCX)

S3 Appendix Detailed sentiment analysis evaluation results of bag-of-embeddings classifiers. (DOCX)
