# Supplementary material for: A versatile framework for resource-limited sentiment articulation, annotation, and analysis of short texts
Source: PLoS One. 2020 Nov 12;15(11):e0242050. doi: 10.1371/journal.pone.0242050 (PMC7660500; doi:10.1371/journal.pone.0242050)
Supplement: S1 Appendix — (DOCX) [file pone.0242050.s001.docx]

**S1 Appendix. Distribution of Texts in the *SentiComments.SR* Verification Corpora across Sentiment Labels.**

Fig. A and B show the distribution of texts across sentiment labels for the movie and the book verification corpus, respectively. Since these corpora were not annotated jointly, as was the case for the main *SentiComments.SR* corpus, we present sentiment label statistics for each annotator individually.

The movie verification corpus has a similar total positive/negative label ratio as the main *SentiComments.SR* corpus. However, the *NS* labels are less numerous than in the larger corpus, while the *M* labels are more frequent, with the exception of control group annotations. The *-1* labels are also slightly more frequent in the verification corpus, whereas the percentage of sarcastic comments is slightly below the one in the main *SentiComments.SR* corpus. Regarding statistical differences between the annotators, it is evident that the control group annotators were generally more likely to use the *+1* label and less inclined to opt for the *M* labels than the rest of the annotators. No major differences are found among the initial group and the experimental group annotators, which is to be expected, since they all relied on the same set of annotation guidelines.

The book verification corpus has a greater proportion of total positive labels than the other two corpora, primarily due to the higher percentage of *+1* and *+NS* labels in it. Consequently, the other four sentiment labels are less frequent in the book verification corpus than they are in the previous two corpora. The percentage of sarcastic texts is similar to the one found in the main *SentiComments.SR* corpus. Regarding statistical differences between the annotators, the control group annotators were more likely to use the *+1* label and less inclined to choose the *+NS* label than the remaining four annotators. They were also less likely to detect sarcasm. As is the case for the movie verification corpus, no major differences are found among the annotators from the initial and the experimental group.

**Figure A. Distribution of Texts in the Movie Verification Corpus across Sentiment Labels for All Annotators.**

**Figure B. Distribution of Texts in the Book Verification Corpus across Sentiment Labels for All Annotators.**
