# Supplementary material for: A versatile framework for resource-limited sentiment articulation, annotation, and analysis of short texts
Source: PLoS One. 2020 Nov 12;15(11):e0242050. doi: 10.1371/journal.pone.0242050 (PMC7660500; doi:10.1371/journal.pone.0242050)
Supplement: S2 Appendix — (DOCX) [file pone.0242050.s002.docx]

**S2 Appendix. Detailed Sentiment Analysis Evaluation Results of Bag-of-Words Classifiers.**

Detailed evaluation results of various options and feature sets applied to linear bag-of-words classifiers are shown in tables for all sentiment classification tasks. Within them, for each set of settings, the label describing the set is in italic. The features and preprocessing options used within that section are listed underneath the label. The best results for each classifier within each section are shown in bold script, if better than their starting baseline, while the selected optimal setting is shaded. Regarding morphological normalizers, (S) denotes a stemmer, and (L) a lemmatizer. CR&EN denotes the character repetition and emoticon normalization procedure.

Table A shows the evaluation results of various bag-of-words/n-grams classifiers and text preprocessing options on the task of polarity detection, where the majority class baseline performance is 0.466. The results indicate that manual text proofing and the proposed normalization techniques have a consistently positive effect on all classifiers. Furthermore, morphological normalization is generally beneficial, with the stemmer by Ljubešić and Pandžić being the optimal choice most frequently. Negation marking is also shown to be useful, but the effects of different negation marking scopes on different classifiers vary widely. In the end, we select the scope of two words after a negation word as the optimal on this task, since this is the only setting that does not diminish the performance of any classifiers. The effects of TFIDF weighting are inconsistent, with a positive impact on Logistic Regression and SVM, yet a noticeably negative one on the remaining three classifiers. Similarly, adding bigram and trigram features has a negligible positive effect on LR and SVM, yet a significantly more negative one on the other classifiers. The cause of these decreases in performance levels linked to TFIDF and higher order n-gram features probably lies in the small size of the dataset and the resulting large data sparsity.

Table B shows the evaluation results of various bag-of-words/n-grams classifiers and text preprocessing options on the task of subjectivity detection, where the majority class baseline performance is 0.743. Similarly to the polarity detection task, the results on subjectivity detection also consistently demonstrate the value of text proofing and the proposed normalization techniques. Morphological normalization is again proven useful, with two similarly good options – the stemmer by Ljubešić and Pandžić and the lemmatizer by Ljubešić et al. For the sake of consistency with the previous task, we selected the stemmer as the optimal choice for further evaluations once again. Contrary to the results on polarity detection, negation marking is found to be detrimental for subjectivity detection. The impact of TFIDF weighting is once more quite inconsistent, with positive effects on MNB, CNB and SVM classifiers, and negative effects on LR and NBSVM. Bigram and trigram features prove to be consistently detrimental to classifier performances on this task.

Table C shows the evaluation results of various bag-of-words/n-grams classifiers and text preprocessing options on the task of four-class sentiment classification (sentiment labels *+1*, *-1*, *M*, and *NS*), where the majority class baseline performance is 0.244. Once again, the results of all classifiers indicate a consistent benefit of text proofing and the proposed normalization techniques. Ljubešić and Pandžić’s stemmer is predominantly the best option for morphological normalization on this task. Due to the presence of polarity labels, negation marking is again found to be useful, with the optimal scope being a single word after a negation term. In contrast, TFIDF weighting is again inconsistent in its effects, being significantly beneficial for the MNB classifier, yet noticeably detrimental for CNB and LR. Bigram and trigram features lead to worse performances of all classifiers on this task.

Table D shows the evaluation results of various bag-of-words/n-grams classifiers and text preprocessing options on the task of full six-class sentiment classification (sentiment labels *+1*, *-1*, *+M*, *-M*, *+NS*, and *-NS*), where the majority class baseline performance is 0.244. As was the case in the previous tasks, text proofing and the proposed normalization techniques are consistently beneficial for all classifiers. Similarly to the subjectivity detection task, two morphological normalization methods stand out – the stemmer by Ljubešić and Pandžić, and the lemmatizer by Ljubešić et al. For the sake of consistency with the previous tasks, we select the stemmer as the optimal option for subsequent evaluations and analyses. Negation marking is also found useful, with the scope of two words after a negation term as the best variant. Once again, TFIDF has mixed effects on classifier performances, with a positive impact on MNB, a negative one on CNB and with little to no effect on LR and SVM. Bigram and trigram features are, once more, shown to have a negative effect on all classifiers.

Table E shows the evaluation results of various bag-of-words/n-grams classifiers and text preprocessing options on the task of sarcasm detection, where the majority class baseline performance is 0. Sarcasm detection is one of the hardest tasks in sentiment analysis, so it is unsurprising that even the options which were consistently beneficial on the previous tasks, such as text proofing and normalization, lead only to small benefits with regard to classifier performances. The effects of morphological normalization, negation marking, TFIDF weighting and higher order n-grams are very inconsistent and often detrimental. This is most likely due to the very small set of sarcastic comments in the corpus, leading to an extremely imbalanced classification problem and increasing the variance of all cross-validation results. We, therefore, concluded that a larger dataset is required to draw confident conclusions regarding classifier performances on the task of sarcasm detection in Serbian, and we did not pursue further experiments regarding this task on our dataset.

**Table A. Evaluation Results of Basic BOW Options on the Task of Polarity Detection.**

| Setting | MNB | CNB | LR | SVM | NBSVM |
| --- | --- | --- | --- | --- | --- |
| *Basic text preprocessing options*  Unigram features | | | | | |
| Original texts | 0.688 | 0.710 | 0.720 | 0.708 | 0.717 |
| Corrected texts | 0.716 | 0.731 | 0.731 | 0.731 | 0.739 |
| Corrected texts + CR&EN | **0.746** | **0.755** | **0.755** | **0.748** | **0.760** |
| *Morphological normalization options*  Corrected texts + CR&EN, unigram features | | | | | |
| (S) Kešelj & Šipka – optimal | 0.760 | **0.775** | 0.757 | 0.757 | **0.772** |
| (S) Kešelj & Šipka – greedy | 0.754 | 0.767 | 0.756 | **0.760** | **0.772** |
| (S) Milošević | 0.761 | 0.773 | 0.756 | 0.755 | **0.772** |
| (S) Ljubešić & Pandžić | **0.765** | 0.773 | **0.758** | 0.756 | **0.772** |
| (L) BTagger – suffix | 0.746 | 0.764 | 0.752 | 0.748 | 0.758 |
| (L) BTagger – prefix + suffix | 0.749 | 0.767 | 0.751 | 0.753 | 0.760 |
| (L) Agić et al. | 0.749 | 0.764 | 0.745 | 0.743 | 0.762 |
| (L) Ljubešić et al. | 0.761 | **0.775** | 0.756 | 0.751 | 0.766 |
| *Negation marking options*  Corrected texts + CR&EN, stemmer – Ljubešić & Pandžić, unigram features | | | | | |
| Negation scope = 1 | 0.762 | 0.776 | 0.762 | 0.759 | 0.774 |
| Negation scope = 2 | 0.767 | 0.776 | 0.762 | 0.758 | **0.782** |
| Negation scope = 3 | **0.769** | **0.779** | 0.759 | 0.753 | **0.782** |
| Negation scope = 5 | 0.764 | 0.775 | 0.761 | 0.761 | **0.782** |
| Negation scope = up to punctuation | 0.754 | 0.771 | **0.767** | **0.766** | 0.778 |
| *Weighting and N-gram features*  Corrected texts + CR&EN, stemmer – Ljubešić & Pandžić, negation scope = 2 | | | | | |
| TFIDF weighting | 0.746 | 0.749 | **0.772** | **0.770** | 0.765 |
| Unigram + bigram features | 0.735 | 0.751 | 0.766 | 0.762 | 0.769 |
| Unigram + bigram + trigram features | 0.720 | 0.732 | 0.760 | 0.759 | 0.768 |

**Table B. Evaluation Results of Basic BOW Options on the Task of Subjectivity Detection.**

| Setting | MNB | CNB | LR | SVM | NBSVM |
| --- | --- | --- | --- | --- | --- |
| *Basic text preprocessing options*  Unigram features | | | | | |
| Original texts | 0.784 | 0.820 | 0.841 | 0.837 | 0.830 |
| Corrected texts | 0.787 | 0.823 | 0.845 | 0.843 | 0.836 |
| Corrected texts + CR&EN | **0.793** | **0.833** | **0.865** | **0.861** | **0.849** |
| *Morphological normalization options*  Corrected texts + CR&EN, unigram features | | | | | |
| (S) Kešelj & Šipka – optimal | 0.807 | 0.853 | 0.870 | 0.865 | 0.858 |
| (S) Kešelj & Šipka – greedy | 0.805 | 0.849 | 0.866 | 0.861 | 0.858 |
| (S) Milošević | **0.808** | 0.850 | 0.868 | 0.868 | 0.861 |
| (S) Ljubešić & Pandžić | 0.807 | **0.855** | 0.871 | 0.863 | **0.864** |
| (L) BTagger – suffix | 0.795 | 0.847 | 0.876 | 0.869 | 0.859 |
| (L) BTagger – prefix + suffix | 0.796 | 0.846 | 0.873 | 0.873 | 0.858 |
| (L) Agić et al. | 0.794 | 0.841 | 0.875 | 0.868 | 0.846 |
| (L) Ljubešić et al. | 0.804 | 0.854 | **0.879** | **0.875** | 0.862 |
| *Negation marking options*  Corrected texts + CR&EN, stemmer – Ljubešić & Pandžić, unigram features | | | | | |
| Negation scope = 1 | 0.806 | 0.851 | 0.867 | 0.863 | 0.856 |
| Negation scope = 2 | 0.803 | 0.847 | 0.869 | **0.864** | 0.861 |
| Negation scope = 3 | 0.800 | 0.847 | 0.865 | 0.861 | 0.862 |
| Negation scope = 5 | 0.799 | 0.844 | 0.861 | 0.857 | 0.857 |
| Negation scope = up to punctuation | 0.797 | 0.841 | 0.864 | 0.858 | 0.859 |
| *Weighting and N-gram features*  Corrected texts + CR&EN, stemmer – Ljubešić & Pandžić, no negation marking | | | | | |
| TFIDF weighting | **0.861** | **0.864** | 0.869 | **0.869** | 0.846 |
| Unigram + bigram features | 0.797 | 0.819 | 0.867 | 0.862 | 0.856 |
| Unigram + bigram + trigram features | 0.791 | 0.811 | 0.859 | 0.856 | 0.842 |

**Table C. Evaluation Results of Basic BOW Options on the Task of Four-class Sentiment Classification.**

| Setting | MNB | CNB | LR | SVM |
| --- | --- | --- | --- | --- |
| *Basic text preprocessing options*  Unigram features | | | | |
| Original texts | 0.460 | 0.544 | 0.567 | 0.566 |
| Corrected texts | 0.498 | 0.562 | 0.579 | 0.584 |
| Corrected texts + CR&EN | **0.523** | **0.602** | **0.616** | **0.617** |
| *Morphological normalization options*  Corrected texts + CR&EN, unigram features | | | | |
| (S) Kešelj & Šipka – optimal | 0.558 | 0.600 | **0.629** | 0.626 |
| (S) Kešelj & Šipka – greedy | 0.556 | 0.599 | 0.626 | 0.624 |
| (S) Milošević | **0.563** | 0.603 | 0.610 | 0.613 |
| (S) Ljubešić & Pandžić | 0.560 | **0.613** | **0.629** | **0.627** |
| (L) BTagger – suffix | 0.545 | 0.599 | 0.621 | 0.620 |
| (L) BTagger – prefix + suffix | 0.542 | 0.600 | 0.618 | 0.615 |
| (L) Agić et al. | 0.526 | 0.590 | 0.615 | 0.610 |
| (L) Ljubešić et al. | 0.558 | 0.603 | 0.622 | 0.624 |
| *Negation marking options*  Corrected texts + CR&EN, stemmer – Ljubešić & Pandžić, unigram features | | | | |
| Negation scope = 1 | 0.560 | **0.616** | **0.640** | **0.631** |
| Negation scope = 2 | **0.562** | 0.613 | 0.632 | 0.628 |
| Negation scope = 3 | 0.555 | 0.609 | 0.626 | 0.628 |
| Negation scope = 5 | 0.550 | 0.609 | 0.630 | 0.625 |
| Negation scope = up to punctuation | 0.542 | 0.603 | 0.625 | 0.623 |
| *Weighting and N-gram features*  Corrected texts + CR&EN, stemmer – Ljubešić & Pandžić, negation scope = 1 | | | | |
| TFIDF weighting | **0.595** | 0.563 | 0.630 | **0.633** |
| Unigram + bigram features | 0.504 | 0.597 | 0.623 | 0.618 |
| Unigram + bigram + trigram features | 0.477 | 0.576 | 0.615 | 0.617 |

**Table D. Evaluation Results of Basic BOW Options on the Task of Six-class Sentiment Classification.**

| Setting | MNB | CNB | LR | SVM |
| --- | --- | --- | --- | --- |
| *Basic text preprocessing options*  Unigram features | | | | |
| Original texts | 0.384 | 0.484 | 0.506 | 0.498 |
| Corrected texts | 0.403 | 0.501 | 0.522 | 0.515 |
| Corrected texts + CR&EN | **0.417** | **0.532** | **0.555** | **0.547** |
| *Morphological normalization options*  Corrected texts + CR&EN, unigram features | | | | |
| (S) Kešelj & Šipka – optimal | 0.441 | 0.536 | **0.557** | 0.554 |
| (S) Kešelj & Šipka – greedy | 0.436 | 0.533 | 0.551 | 0.550 |
| (S) Milošević | 0.443 | 0.539 | 0.556 | 0.553 |
| (S) Ljubešić & Pandžić | **0.444** | 0.540 | **0.557** | 0.547 |
| (L) BTagger – suffix | 0.435 | 0.538 | 0.549 | 0.555 |
| (L) BTagger – prefix + suffix | 0.437 | 0.537 | 0.554 | **0.556** |
| (L) Agić et al. | 0.421 | 0.526 | 0.548 | 0.541 |
| (L) Ljubešić et al. | 0.442 | **0.548** | **0.557** | 0.555 |
| *Negation marking options*  Corrected texts + CR&EN, stemmer – Ljubešić & Pandžić, unigram features | | | | |
| Negation scope = 1 | 0.443 | **0.547** | 0.562 | 0.560 |
| Negation scope = 2 | **0.445** | 0.546 | **0.566** | 0.561 |
| Negation scope = 3 | 0.442 | 0.539 | 0.563 | 0.561 |
| Negation scope = 5 | 0.439 | 0.541 | 0.563 | **0.562** |
| Negation scope = up to punctuation | 0.436 | 0.534 | 0.565 | 0.560 |
| *Weighting and N-gram features*  Corrected texts + CR&EN, stemmer – Ljubešić & Pandžić, negation scope = 2 | | | | |
| TFIDF weighting | **0.531** | 0.510 | **0.569** | **0.569** |
| Unigram + bigram features | 0.409 | 0.516 | 0.556 | 0.554 |
| Unigram + bigram + trigram features | 0.399 | 0.504 | 0.552 | 0.544 |

**Table E. Evaluation Results of Basic BOW Options on the Task of Sarcasm Detection.**

| Setting | MNB | CNB | LR | SVM | NBSVM |
| --- | --- | --- | --- | --- | --- |
| *Basic text preprocessing options*  Unigram features | | | | | |
| Original texts | 0 | 0 | 0.040 | 0.059 | 0.038 |
| Corrected texts | 0 | 0 | 0.030 | 0.043 | 0.055 |
| Corrected texts + CR&EN | 0 | 0 | **0.096** | **0.124** | **0.070** |
| *Morphological normalization options*  Corrected texts + CR&EN, unigram features | | | | | |
| (S) Kešelj & Šipka – optimal | 0 | **0.014** | **0.167** | 0.175 | 0.052 |
| (S) Kešelj & Šipka – greedy | 0 | 0 | 0.104 | 0.137 | 0.040 |
| (S) Milošević | 0 | 0 | 0.121 | 0.141 | 0.060 |
| (S) Ljubešić & Pandžić | 0 | 0 | 0.112 | **0.186** | 0.054 |
| (L) BTagger – suffix | 0 | 0 | 0.095 | 0.115 | 0.038 |
| (L) BTagger – prefix + suffix | 0 | 0 | 0.107 | 0.129 | 0.053 |
| (L) Agić et al. | 0 | **0.014** | 0.082 | 0.098 | **0.079** |
| (L) Ljubešić et al. | 0 | 0 | 0.100 | 0.115 | 0.072 |
| *Negation marking options*  Corrected texts + CR&EN, no morphological normalization, unigram features | | | | | |
| Negation scope = 1 | 0 | 0 | 0.029 | 0.095 | 0.070 |
| Negation scope = 2 | 0 | 0 | 0.014 | 0.079 | **0.072** |
| Negation scope = 3 | 0 | 0 | 0.031 | 0.081 | 0.045 |
| Negation scope = 5 | 0 | 0 | 0.014 | 0.105 | 0.057 |
| Negation scope = up to punctuation | 0 | 0 | 0.046 | 0.108 | 0.058 |
| *Weighting and N-gram features*  Corrected texts + CR&EN, no morphological normalization, no negation marking | | | | | |
| TFIDF weighting | **0.156** | **0.149** | 0.071 | 0.081 | 0.017 |
| Unigram + bigram features | 0 | 0.015 | 0.015 | 0.030 | 0 |
| Unigram + bigram + trigram features | 0 | 0 | 0 | 0 | 0 |
