# Supplementary material for: A versatile framework for resource-limited sentiment articulation, annotation, and analysis of short texts
Source: PLoS One. 2020 Nov 12;15(11):e0242050. doi: 10.1371/journal.pone.0242050 (PMC7660500; doi:10.1371/journal.pone.0242050)
Supplement: S3 Appendix — (DOCX) [file pone.0242050.s003.docx]

**S3 Appendix. Detailed Sentiment Analysis Evaluation Results of Bag-of-Embeddings Classifiers.**

Detailed evaluation results of various options and feature sets applied to linear bag-of-embeddings SVM classifiers are shown in Table A for all sentiment classification tasks. Within them, for each set of settings, the label describing the set is in italic. The features and preprocessing options used within that section are listed underneath the label. The best results within each section are shown in bold script, if better than their starting baseline, while the selected optimal setting is shaded. Regarding morphological normalizers, (S) denotes a stemmer, and (L) a lemmatizer. CR&EN denotes the character repetition and emoticon normalization procedure.

We first explore the effects of text proofing and normalization techniques in this setup. We use 100-dimensional embeddings generated with a context window size of 10. All other *word2vec* parameters are kept at the default *gensim* settings for the *skip-gram* architecture. Texts from both the *SentiComments.SR* corpus and the *srWaC* corpus are lowercased. The results show that manual proofing, and character repetition and emoticon normalization are beneficial to classification performances on all tasks. Next, we consider morphological normalization methods. To this end, separate sets of word embeddings are produced for each morphologically normalized variant of the *srWaC* corpus. As is the case for bag-of-words models, morphological normalization once again generally improves classification performances, with Ljubešić and Pandžić’s stemmer proving to be the optimal choice in this setup, as well.

We then evaluate the impact of word embedding dimensionality and window size by considering dimensions ranging from 100 to 1000 and two window size options – 5 and 10. This set of embeddings is trained on the *srWaC* corpus, which was processed by Ljubešić and Pandžić’s stemmer. We find that classification performances generally improve as the dimensionality and window size are increased. This effect is least pronounced on the task of subjectivity detection, and most apparent on the tasks of four-class and six-class sentiment classification. We obtain the best results with the largest embedding dimensionality and window size – 1000 and 10, respectively.

Finally, using these parameters, we consider two other options. Firstly, we implement the negation-marking technique by labeling a single token after each negation word in the preprocessed and stemmed *srWaC* and generating negation-marked *word2vec* embeddings from such a corpus. Optimal task-specific negation scopes, determined in the evaluation of bag-of-words models, are used for the *SentiComments.SR* texts in this setup, as well. We find that this approach yields only a slight increase in the performance on the polarity detection task, at the cost of a slight decrease on the four-class sentiment classification task. Afterwards, we experiment with combining word embedding-based features with bag-of-words features, using the task-specific optimal settings determined in the evaluation of bag-of-words models. This setup, particularly when using negation marking for BOW features, proves to be noticeably better than the plain embeddings-based one on all tasks except polarity detection, where no benefit is observed. However, negation-marked embeddings actually lead to slightly worse performances than regular embeddings in this setup.

**Table A. Evaluation Results of Bag-of-Embeddings SVM Models.**

| Setting | Task results | | | |
| --- | --- | --- | --- | --- |
|  | Polarity | Subjectivity | Four-class | Six-class |
| *Basic text preprocessing options*  Averaged word2vec skip-gram features, dimensionality = 100, window size = 10 | | | | |
| Original texts | 0.710 | 0.836 | 0.516 | 0.462 |
| Corrected texts | 0.720 | 0.842 | 0.536 | 0.474 |
| Corrected texts + CR&EN | **0.745** | **0.858** | **0.559** | **0.506** |
| *Morphological normalization options*  Corrected texts + CR&EN, averaged word2vec skip-gram features, dimensionality = 100, window size = 10 | | | | |
| (S) Kešelj & Šipka – optimal | 0.744 | 0.859 | 0.559 | 0.509 |
| (S) Kešelj & Šipka – greedy | 0.745 | 0.864 | 0.548 | 0.494 |
| (S) Milošević | 0.740 | 0.858 | 0.562 | 0.499 |
| (S) Ljubešić & Pandžić | **0.761** | **0.866** | **0.566** | **0.511** |
| (L) BTagger – suffix | 0.748 | 0.858 | 0.562 | 0.510 |
| (L) BTagger – prefix + suffix | 0.746 | 0.861 | 0.557 | 0.502 |
| (L) Agić et al. | 0.747 | 0.851 | 0.553 | 0.498 |
| (L) Ljubešić et al. | 0.741 | 0.857 | 0.564 | 0.506 |
| *Embedding dimensionality and window size*  Corrected texts + CR&EN, stemmer – Ljubešić & Pandžić, averaged word2vec skip-gram features | | | | |
| Dimensionality = 100, window size = 5 | 0.757 | 0.861 | 0.564 | 0.512 |
| Dimensionality = 100, windows size = 10 | 0.761 | 0.866 | 0.566 | 0.511 |
| Dimensionality = 300, window size = 5 | 0.767 | 0.865 | 0.604 | 0.533 |
| Dimensionality = 300, window size = 10 | 0.770 | 0.869 | 0.599 | 0.535 |
| Dimensionality = 500, window size = 5 | 0.775 | 0.872 | 0.614 | 0.543 |
| Dimensionality = 500, window size = 10 | 0.777 | 0.870 | 0.616 | 0.546 |
| Dimensionality = 1000, window size = 5 | **0.783** | 0.871 | 0.626 | **0.558** |
| Dimensionality = 1000, window size = 10 | **0.783** | **0.873** | **0.628** | 0.557 |
| *Other settings*  Corrected texts + CR&EN, stemmer – Ljubešić & Pandžić, averaged word2vec skip-gram features, dimensionality = 1000, window size = 10 | | | | |
| Negation-marked embeddings | **0.788** | / | 0.622 | 0.558 |
| Adding BOW features without negation marking | 0.780 | **0.885** | **0.655** | 0.576 |
| Adding BOW features with task-specific optimal negation marking | 0.783 | **0.885** | **0.655** | **0.586** |
| Negation-marked embeddings + BOW features with task-specific optimal negation marking | 0.778 | / | 0.652 | 0.585 |
